# Efficacy of HER2-targeted therapies in localized urothelial carcinoma: a systematic review and meta-analysis

**DOI:** 10.1007/s00345-026-06868-2

**Published:** 2026-11-14

**Authors:** Agata Suleja, Deborah Bret, Marcin Miszczyk, Wen Dong, Xi Zhou, Ekaterina Laukhtina, Bernhard Englinger, Paweł Rajwa, Pierre I. Karakiewicz, Vérane Achard, Bernadett Szabados, Eva Compérat, Jonathan E. Rosenberg, Pixiao Zhou, Shahrokh F. Shariat

**Affiliations:** 1 Department of Urology, Comprehensive Cancer Center, Vienna General Hospital, Medical University of Vienna, Währinger Gürtel 18-20, 1090 Vienna, Austria; 2 Collegium Medicum, WSB University, Dąbrowa Górnicza, Poland; 3 Department of Oncology, Changde Hospital, Xiangya School of Medicine, Central South University (The First People’s Hospital of Changde City), Changde, China; 4 Center for Cancer Research, Medical University of Vienna, Vienna, Austria; 5 Division of Surgery and Interventional Sciences, University College London, University College London Hospital, London, UK; 6 Department of Urology, Medical University of Silesia, Zabrze, Poland; 7 Cancer Prognostics and Health Outcomes Unit, Division of Urology, University of Montréal Health Center, Montréal, Québec Canada; 8 Department of Radiotherapy, Institut Bergonié, Bordeaux, France; 9 University of Geneva, Geneva, Switzerland; 10 Barts Experimental Cancer Medicine Centre, Barts Cancer Institute, Queen Mary University of London, London, UK; 11 Department of Urology, University College London Hospital NHS Foundation Trust, London, UK; 12 Department of Pathology, Medical University of Vienna, General Hospital Vienna, Vienna, Austria; 13 Genitourinary Oncology Service, Department of Medicine, Memorial Sloan Kettering Cancer Center, New York, NY USA; 14 Department of Urology, Weill Cornell Medical College, New York, NY USA; 15 Department of Urology, University of Texas Southwestern, Dallas, TX USA; 16 Department of Urology, Second Faculty of Medicine, Charles University, Prague, Czech Republic; 17 Hourani Center for Applied Scientific Research, Al-Ahliyya Amman University, Amman, Jordan; 18 Karl Landsteiner Institute of Urology and Andrology, Vienna, Austria

**Keywords:** HER2, Disitamab vedotin, Antibody–drug conjugate, Urothelial carcinoma, Neoadjuvant therapy

## Abstract

**Background:**

HER2-targeted antibody–drug conjugates were recently approved in China for treatment of advanced or metastatic urothelial carcinoma (UC). We summarise emerging evidence supporting use of HER2-targeted regimens in patients with localized disease.

**Methods:**

We searched MEDLINE, Embase, Web of Science, CENTRAL through March 29, 2026, and SinoMed, Wanfang and CNKI through October 14, 2025 to identify studies evaluating HER2-targeted therapies, in patients with localized UC. Random-effects meta-analyses pooled response rates, including pathological complete response (pCR). Clinical complete response (cCR) was stratified by response definition. Heterogeneity was assessed using Cochrane's Q test. Risk-of-bias (RoB) was assessed using ROBINS-I tool. The study was registered prospectively (CRD420250650326).

**Results:**

Thirty-three studies comprising 970 patients were included (17 prospective); 30 tested disitamab vedotin (DV). In 13 studies testing DV-containing regimens as neoadjuvant therapy, the pooled pCR rate was 48% (95% CI 39–58%, N = 398; 54% among surgery-evaluable patients). Evidence was insufficient to establish an association between HER2 expression and pCR (risk ratio [RR] 0.82, 95% CI 0.52–1.29; p = 0.38; N = 72). In bladder-sparing studies, cCR was 65% (95% CI 44–82%) with definitions requiring complete absence of residual tumor and 54% (95% CI 41–67%) with definitions permitting residual Ta/Tis disease. Among included trials using DV, rate of grade ≥3 adverse events did not exceed 35% and was lower than that reported in studies evaluating other HER2-targeted agents. Major RoB concerns regarded outcome measurement as none of included trials was blinded.

**Conclusions:**

DV-containing regimens show promising activity as neoadjuvant therapy in patients with bladder cancer. Current evidence is insufficient to establish an association between HER2 expression and response. Emerging data indicate possible role in bladder-sparing setting with low rates of toxicity and no G5 safety signals, although heterogeneous cCR definitions limit interpretation.

**Supplementary Information:**

The online version contains supplementary material available at https://doi.org/10.1007/s00345-026-06868-2.

## Background

Neoadjuvant systemic therapy is standard of care (SOC) in patients with muscle-invasive bladder cancer (MIBC). This approach was developed with chemotherapy [1], but even with modern platinum-based regimens, the rates of pathologic complete response (pCR) are modest [2]. The NIAGARA trial introduced a practice-changing “sandwich” approach, adding durvalumab to standard-of-care chemotherapy, while CheckMate 274 [3] and AMBASSADOR [4] proved efficacy of adjuvant immune checkpoint inhibitor (ICI) treatment with nivolumab and pembrolizumab, respectively. ICIs with chemotherapy also provide favorable results in the neoadjuvant setting, with over twofold higher rates of pCR compared to chemotherapy alone, though with an uncertain impact on mortality [5]. Recently, a major breakthrough was introduced by the KEYNOTE−905 study for cisplatin-ineligible patients. Enfortumab vedotin (EV), an antibody–drug conjugate (ADC), combined with pembrolizumab (P) led to pCR in over half of the patients and a twofold improvement in overall survival (OS), followed by a FDA approval of EV+P as perioperative therapy for platinum-ineligible patients [6]. Additionally results of treatment with EV+P in cisplatin-eligible population showed over 20% improvement in rate of pCR compared with chemotherapy [7]. There is a clear shift towards molecularly targeted ADCs or ICIs in MIBC treatment paradigms; however, these highly molecularly precise agents are being used in all-comers, with little personalization of treatment beyond “cisplatin-eligibility”.

The mutation in the ERBB2 gene encoding human epidermal growth factor receptor 2 (HER2), as well as simply overexpression of the receptor has been associated with tumorigenesis, disease progression, and impaired antitumor immune responses [8–10]. HER2-directed therapies, including trastuzumab or pertuzumab, are well-established in breast, gastric and esophageal cancers, though their success in urothelial carcinoma (UC) setting is limited [11–13]. Recently, a HER2 targeted agent disitamab vedotin (DV) was approved in China for first-line therapy in metastatic and advanced UC, following the results of the RC48-C016 trial [14], while the DESTINY-PanTumor02 trial led to the approval of trastuzumab deruxtecan (T-DXd) in patients with unresectable or metastatic HER2-positive (IHC3 +) solid tumors failed after prior systemic therapy, including metastasized UC [15]. In this review, we aimed to summarize the emerging evidence supporting the efficacy of HER2-targeted regimens in the setting of localized UC including bladder and upper tract UC.

## Methods

### Protocol and search strategy

The study was prospectively registered at PROSPERO (CRD420250650326) and adhered to the Preferred Reporting Items for Systematic Reviews and Meta-analyses (PRISMA) statement, including the PRISMA 2020 checklist (Supplementary File 1) and PRISMA for Abstracts checklist (Supplementary File 2) [16]. The inclusion criteria were defined using the Population, Intervention, Comparator, Outcome, and Study Design (PICOS) framework (Supplementary File 3).

We searched MEDLINE (via PubMed), Embase, Web of Science, and Cochrane Central Register of Controlled Trials (CENTRAL) through March 29, 2026. Additionally, the top 200 hits were retrieved using Google Scholar search engine. Chinese databases: SinoMed, Wanfang and China National Knowledge Infrastructure (CNKI) were searched on October 14, 2025. The search was performed using a predefined search strategy which can be found in Supplementary File 4. The deduplication and screening process were performed using Rayyan software (Qatar Computing Research Institute, Doha, Qatar) [17]. Abstract screening, as well as appraisal of full-text articles was performed independently by two authors (Supplementary File 5). All conflicts were resolved through mediation.

### Study selection

We included studies testing HER2-targeted regimens as perioperative (neoadjuvant, adjuvant) or organ-sparing treatment in patients with UC reporting on pathological complete response (pCR), clinical complete response (cCR), pathological downstaging (pDS), objective response rate (ORR), bladder preservation rate, and safety of treatment. Both retrospective and prospective studies were eligible. We excluded studies incorporating various solid tumors, which did not separately report outcomes for UC patients.

### Data extraction

Data on the sample size, disease setting, HER2 expression, treatment, treatment outcomes (pCR, cCR, pDs, ORR, bladder-preservation rate) and rate of treatment related adverse events (TRAEs) were extracted independently by two authors using a predefined data extraction sheet. For comparative trials, only data for HER2-targeted therapies were extracted. Conflicts were resolved through mediation. Multiple reports of the same trial were treated as a single study; where overlap could not be confirmed or excluded, both reports were retained and examined in sensitivity analyses.

### Risk of bias assessment

The risk of bias (RoB) for each study was assessed independently by two authors using the ROBINS-I tool [18]. Conflicts were resolved through mediation.

### Statistical analysis

All pooled estimates used all treated patients as the denominator, including patients without surgery or response assessment as non-responders. For pCR and pDS sensitivity analysis included only surgery evaluable patients. Neoadjuvant pCR was stratified by cisplatin eligibility. Further sensitivity analyses excluded potentially overlapping cohorts and were restricted to DV combined with ICI and to full-text publications. Subset analyses based on HER2 expression (denominators reported for each HER2 expression group) and localization of the disease were performed. In bladder-sparing studies, cCR rates were stratified by cCR definition. Random-effects models using inverse-variance Paule-Mandel estimator for τ^2^ calculation were used to pool log-transformed proportions across studies. Back-transformed outcomes were presented on forest plots with corresponding 95% confidence intervals. A continuity correction of 0.5 was applied to both the numerator and denominator in all studies. Between-study heterogeneity was assessed using Cochran’s Q test and *I*^2^. Leave-one-out sensitivity analyses were performed to identify influential studies. Publication bias was evaluated using funnel plots and Egger’s precision-effect test in analyses with >10 studies. Statistical analysis was performed using R software v4.5.1 (R Foundation for Statistical Computing, Vienna, Austria), and the “meta” package.

## Results

### Included studies

Out of 4701 individual records screened, we identified 33 studies (N = 970) published between 2015 and 2026 that were eligible for systematic review, including eight prospective non-randomized comparative trials (N = 252) [19–26], nine prospective single arm trials (N = 286) [22, 27–34], and 16 retrospective studies (N = 432) [34–48]. Majority of the studies were conducted in China (N = 802) [20–22, 24, 26–39, 41–51] and three were performed in North America (N = 168) [19, 23, 25]. The included studies were relatively small, with a median cohort size of 20 participants (interquartile range [IQR] 15–34), including only two trials with >100 participants [25, 36]. Median age was 67.5 (interquartile range [IQR] 65–69.25). Patients were treated mainly with HER2-targeted ADC–DV alone or combined with toripalimab, tislelizumab, cadonilimab or pembrolizumab. Other drugs included lapatinib, trastuzumab and DN24-02.

#### Risk of bias analysis

The summary of RoB analysis is presented in Supplementary File 6. According to the ROBINS-I tool, 24 studies were assessed as moderate [20–22, 25–29, 32–35, 37, 39–45, 48, 50–52], eight as serious [19, 23, 24, 36, 38, 46, 47, 53], and one as having critical RoB [30]. The main source of bias domains was outcome measurement as none of the included studies were blinded, outcome measures were partially subjective, and there were missing data. Many included studies were available only as conference abstracts and reported immature data. Additionally, patient selection was an important domain of concern in many cases due to missing robust definitions of inclusion criteria and/or retrospective study design.

### Neoadjuvant setting

#### Bladder cancer

**Fig. 1:**
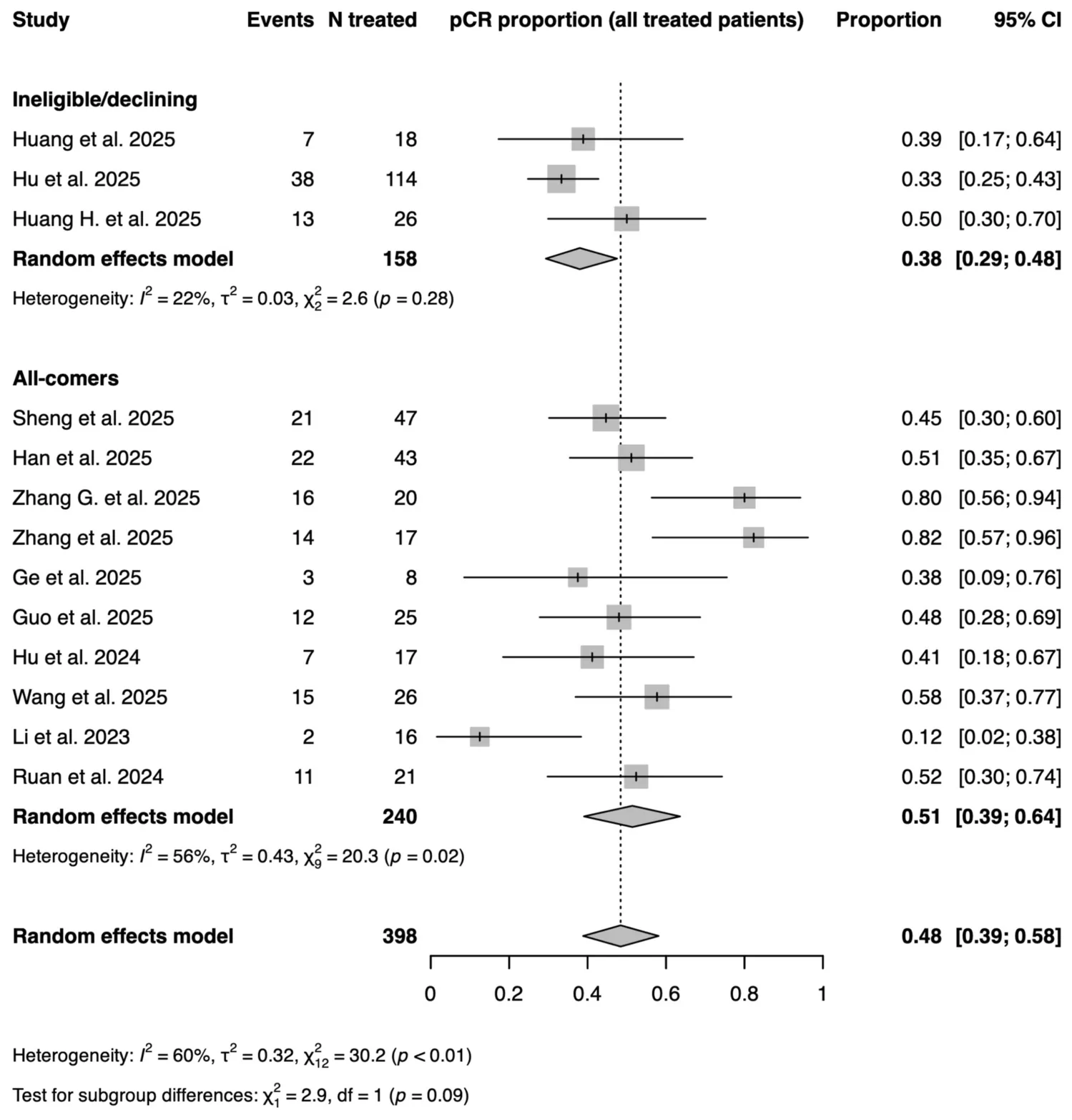
Forest plot of rates of pCR across included neoadjuvant bladder cancer studies using disitamab vedotin **a** all comers population **b** cisplatin ineligible

The efficacy of neoadjuvant HER2-targeted therapies was assessed in 15 studies [19, 27–30, 36, 38–41, 41–43, 48, 51, 52] (N = 419). Majority used a combination of DV and ICI (toripalimab/tislelizumab/cadonilimab or pembrolizumab), as summarized in Table 1. Only study by Huang et al. evaluated the efficacy of DV alone [27], and one study combined DV with gemcitabine [52]. Among 13 studies evaluating DV-containing regimens as HER2-targeted therapy, the pooled pCR rate was 48% (95% CI 39–58%, N = 398) with all treated patients in the denominator (Fig. 1), and 54% (95% CI 45–62%, N = 350) among surgery-evaluable patients (Suppl. Figure 1a).

**Table 1:** Studies with the HER2-targeted therapies in the neoadjuvant setting

1st Author, Year	Study Design (Study Identifier)	Inclusion criteria	Publication type	N of Patients	N of Males	Median Age	Median FU [mo]	Treatment	HER2 expression				pCR [%]	pDS [%]	Additional outcomes	TRAE [%]	TRAE G≥3 [%]	Most common TRAEs
									3+	2+	1+	0						
*MIBC*																		
Huang et al. 2025 [1]	Phase II, SA (ChiCTR2300068270)	treatment-naïve HER2- positive (IHC 2+, 3 +) MIBC (cT2-T4a, N0-1, M0; ECOG PS score 0–1), refuse or unfit for cisplatin-based NAC	Abstract	17	12	71	10.1	DV (4 cycles)	9	8			41.18		PaIR = 64.71%	88.27	0	Alopecia, AST and ALT elevation, PNP
Sheng X. et al. 2025 [2]	Phase II, SA (RC48-C017/ NCT05297552)	treatment-naïve HER2-positive (IHC ≥1) MIBC (cT2-4aN0-1M0) eligible for RC + PLND	Abstract	47				DV + Toripalimab (6 cycles + up to 20 cycles adj. Toripalimab)	15	27	5		63.6	75.8	PaIR = 75.8% 1-y EFS = 89.5%		15.9	Alopecia, AST, ALT, GGTP increased, PNP
Han S. et al. 2025 [3]	Phase II, SA (NCT06074484)	treatment-naïve HER2- positive (IHC 1+, 2+, 3 +) MIBC (T2-T4a, N0-1, M0; ECOG PS score 0–1)	Abstract	43	35	65	11.3	DV + Cadonilimab (6 cycles + 6 (14 Cadonilimab) cycles adj	31 (2/3 +)				64.71	76.47		90.7		Fever, rash, bone marrow hypocellular, ALT elevation, immune-mediated myocarditis and pneumonitis
Hu et al. 2025 [4]	Retrospective	treatment-naïve BC, no distant metastases, no UTU; cisplatin ineligible	Article	102			5.5	DV + Tislelizumab/ Toripalimab (2–6 cycles)	74 (2/3 +)		28 (0/1 +)		37.3	75.5	1-y DFS= 97.4%			
Li et al. 2025 [5]	Phase II, SA	HER2 positive (HER2 IHC1+, 2+, or 3 +) cT2a-4aN0M0, unfit for cisplatin-based NAC	Abstract	28		68	11.8	DV + Toripalimab (4 cycles)					93.75**		ORR = 96.4%	75	14.3	Cardiac tachycardia, hepatic dysfunction
Narayan et al. 2016 [6]	Phase I/II	HER2 positive (IHC >1 +), T2-T4, N0, M0, ECOG 0–1	Article	6	4	62.5		Lapatinib, +Gem/Cis ( 4 cycles)	3 (HER2 ≥1 +)			3	17	33		100	100	Diarrhea, nausea/vomiting, thrombocytopenia
Huang H. et al. 2025 [7]	Phase II, SA (NCT05723991)	MIBC (T2-4N0M0) HER2 positive (2/3 +), unfit for cisplatin-based NAC	Article	26	25	62	16.9	DV + Gem/Cis (3/4 cycles)	6	20			59		ORR = 100% 3 cycles pCR = 31% 4 cycles pCR = 69%		0	AST, ALT elevation, decreased appetite, alopecia, PNP
Zhang G. et al. 2025 [8]	Retrospective	locally advanced HER2 positive MIBC	Abstract	20		65		DV + Toripalimab					80	100	ORR = 100% mPFS=4.5 mo		5	Nausea, alopecia, rash
Zhang et al. 2025 [9]	Retrospective	treatment-naïve HER2-positive MIBC (cT2-4aN0M0)	Article	17	11	72.94 (mean)		DV + Toripalimab/ Tislelizumab (at least 3 cycles)	7	9	1	0	82.35	94.12	ORR = 94.12%		5.71	Decreased apetite, asthenia, pruritus, alopecia, leukopenia
Ge et al. 2025* [10]	Retrospective	Primary UC confirmed by histopathology; ≥1 DV treatment; Available response assessments;	Article	8				DV + Tislelizumab/ Tolipalimab (at least 1 cycle)	8 (2/3 +)				37.5		ORR = 100%			Hypoesthesia, elevated ALT, AST
Guo et al. 2025 [11]	Retrospective (2025R0556)	localized MIBC (cT2-4aN0-1M0), received ≥4 neoadjuvant therapy with DV + PD-1 inhibitor followed by RC	Article	25	21	69	17	DV + Tislelizumab/ toripalimab/ pembrolizumab (at least 4 cycles)	13	10	1	1	48	88	1-yr DFS = 91.5% 1-yr OS = 100%	92	16	PNP, rash, ALT and AST elevated
Hu et al. 2024 [12]	Retrospective	T2-4aN0-2M0 MIBC	Article	17	16	65		DV + Tislelizumab	13 (2/3 +)		3	1	41.2	58.8			35.3	ALT and AST elevated, PNP, anemia
Wang et al. 2025 [13]	Retrospective	T1-4N0-2M0 UC	Article	26	19	66		DV + ICIs (PD-1)	22 (2/3 +) no FISH		4 (0/1 +)		57.7				0	PNP, rash
Li et al. 2023 [14]	Retrospective	T2-3N0M0 UC	Article	16	15	66.12 (mean)		DV + Tislelizumab	3	6	6	1	25	62.5	ORR = 75% cCR=31.25%	100	31.3	Rash, hypothyroidism, hyperglycemia
Ruan et al. 2024 [15]	Retrospective	high-risk or very high-risk NMIBC (Tis-T1N0M0), and MIBC (T2-4N0M0)	Article	21	16	65 (mean)	9	DV + ICIs (PD-1)	6	10	5	0	52.4	80.95	ORR = 85.7%	100	14.3	Hypertriglyceridemia, hyperglycemia, renal dysfunction
*UTUC*																		
Bao et al. 2024 [16]	Phase II, SA	locally advanced UTUC eligible to undergo RNU, regardless HER2 expression	Abstract	14	8	69	65w	DV + Tislelizumab (4 cycles)					21.43		ORR = 78.57%		7.14	
Chen H. et al. 2025 [17]	Pilot (NCT05837806)	cT1-N0-M0 UTUC HER2 (IHC ≥1 +)	Abstract	15	12	71	117 d	DV + Tislelizumab (3 cycles)	2	9	3		25				10.5	Fatigue, PNP, alopecia

*MIBC* muscle-invasive bladder cancer, *UTUC* upper-tract urothelial carcinoma, *N* number, *FU* follow-up, *mo* month, *pCR* pathological complete response, *pDS* pathological down-staging, *TRAE* treatment-related adverse events, *IHC* immunohistochemistry, *NAC* neoadjuvant chemotherapy, *RC* + *PLND* radical cystectomy with pelvic lymph node dissection, *DV* Disitamab vedotin, *ICI* immune-checkpoint inhibitor, *PaIR* pathologic muscle-invasive response rate (≤ ypT1N0), *EFS* event-free survival, *DFS* disease-free survival, *ORR* objective response rate, *mPFS* median progression-free survival, *RNU* radical nephroureterectomy, *AST* aspartate aminotransferase, *ALT* alanine aminotransferase, *PNP* peripheral sensory neuropathy, *GGTP* gamma-glutamyl transferase, *adj*. adjuvant

^*^part of the bigger trial in the metastatic setting

^**^measured only among patients with cCR

In predominantly cisplatin-eligible all-comers cohort the pooled pCR rate was 51% (95% CI 39–64%, N = 240) [28, 29, 38–43, 48, 51], and 38% in cisplatin-ineligible or cisplatin-declining cohorts (95% CI 29–48%, N = 158) (Fig. 1) [27, 36, 52]. There was no evidence for significant differences between subgroups (p = 0.09) or publication bias (p = 0.19; Suppl. Figure 1b); there was no significant heterogeneity in the cisplatin ineligible/declining cohort (I^2^ = 22%, p = 0.28), however the heterogeneity was significant in cisplatin-eligible population (I^2^ = 56%, p = 0.02). Leave-one-out analysis revealed heterogeneity was mainly attributable to Li et al., in which only 8 out of 16 treated patients underwent radical cystectomy (Suppl. Figure 1c) [41]. After excluding Li et al. the pooled pCR rate in all-comers cohort was 54% (45–64%; N = 224), and the heterogeneity was no longer significant (I^2^ = 39%, p = 0.11) (Supplementary Fig. 1d). In a sub-analysis of 11 studies evaluating only DV combined with ICI the pooled pCR rate was 49% (95% CI 38–61%, N = 354) (Suppl. Figure 2). DV monotherapy and DV combined with gemcitabine were each evaluated in a single study, with pCR in 7/18 (39%) and 13/26 (50%) treated patients, respectively [27, 52]. In additional sub-analysis of studies published as full-text articles the pooled pCR rate was 46% (95% CI 34–59%, N = 270) (Suppl. Figure 3). Excluding potentially overlapping cohorts from the same institutions [28, 40, 41, 43, 51], individually or in combinations, the pooled pCR rate remained stable (range 48–52%; Suppl. Figure 4).

Evidence was insufficient to establish an association between HER2 expression and pCR. The pooled pCR rate was 54% (95% CI 24–81%, N = 20) in HER2 0/1+ tumors and 53% (95% CI 43–63%, N = 134) in HER2 2/3+ tumors, with no evidence for subgroup differences (p = 0.94). The pooled risk ratio (RR) for pCR in patients with HER2 2/3+ expression (N = 52) compared with those with 0/1+ HER2 expression (N = 20) was 0.82 (95% CI 0.52–1.29; p = 0.38) [29, 40, 42] (Supplementary Fig. 5).

**Fig. 2:**
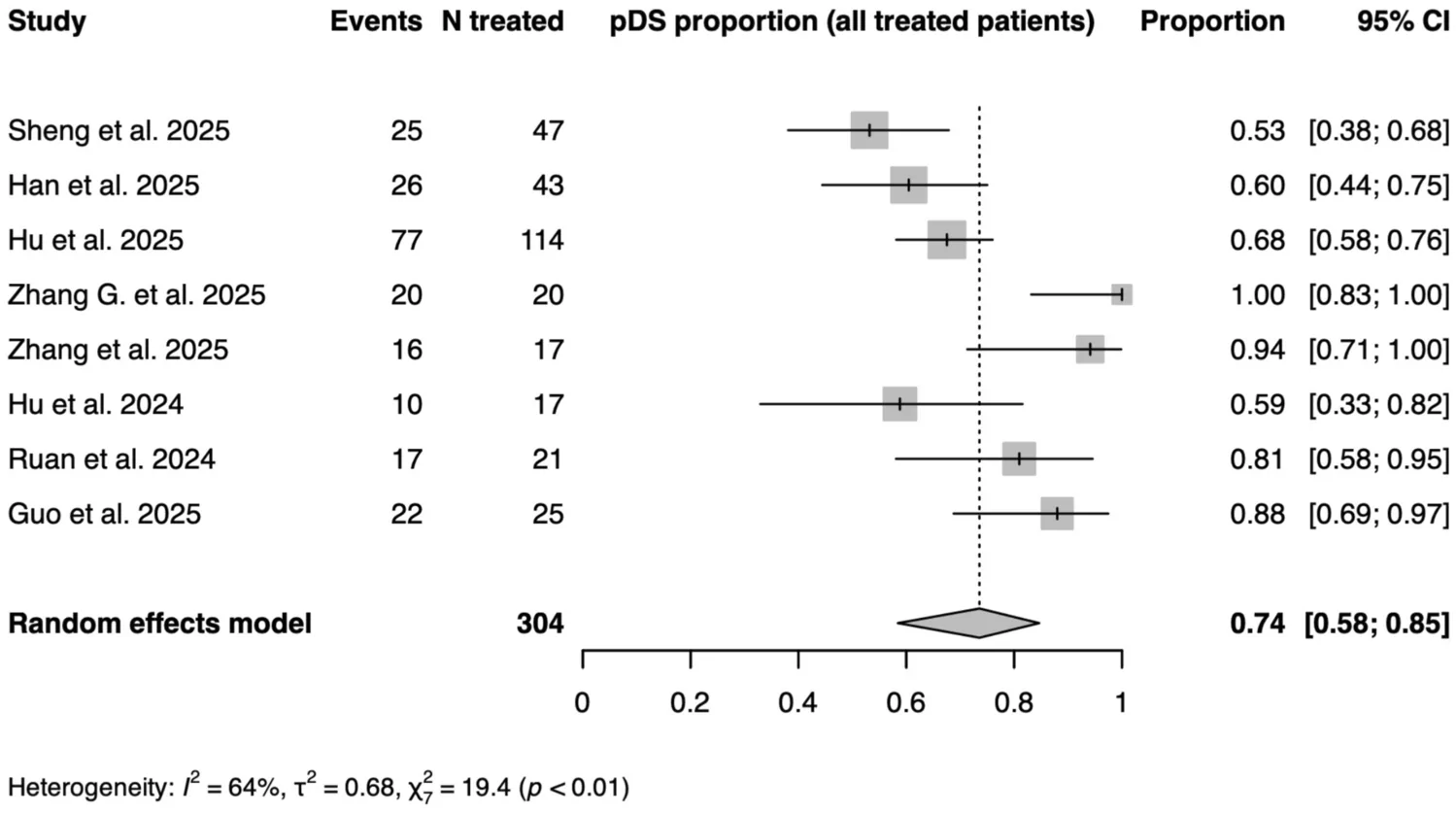
Forest plot of rates of pathological down-staging across included neoadjuvant bladder cancer studies

pDS was evaluated in eight studies using DV+ICI, with an estimated pooled rate of 74% (95% CI 58–85%, N = 304) (Fig. 2) [28, 29, 36, 38, 39, 42, 43]. There was significant heterogeneity (*I*^*2*^=64%, p<0.01), however leave-one-out analysis revealed that exclusion none of the studies markedly reduce the heterogeneity (Suppl. Figure 6a). Among surgery-evaluable patients pDS rate was 78% (95% CI 69–85%, N = 269) (Suppl. Figure 6b) and there was no significant heterogeneity (*I*^*2*^=30%, p = 0.19), suggesting that heterogeneity in all-treated analysis reflected differences in proportion of patients undergoing sugery. One study was excluded from meta-analysis [30] due to critical RoB, as pCR was assessed only in patients who achieved cCR.

The most common TRAEs among included trials using DV and immunotherapy were alopecia, elevation of liver enzymes, and peripheral sensory neuropathy (PNP). All of them were generally manageable and there was no treatment-related deaths reported. The rate of grade 3 or worse TRAEs varied from 5 to 35%. [27–30, 36, 38–42, 48, 51, 52].

One study evaluated another HER2-directed therapy—lapatinib [19]. After enrolling six patients, the trial was prematurely closed due to a low pCR rate (17%) and the occurrence of serious TRAEs in all participants.

#### Upper-tract urothelial cancer

**Fig. 3:**
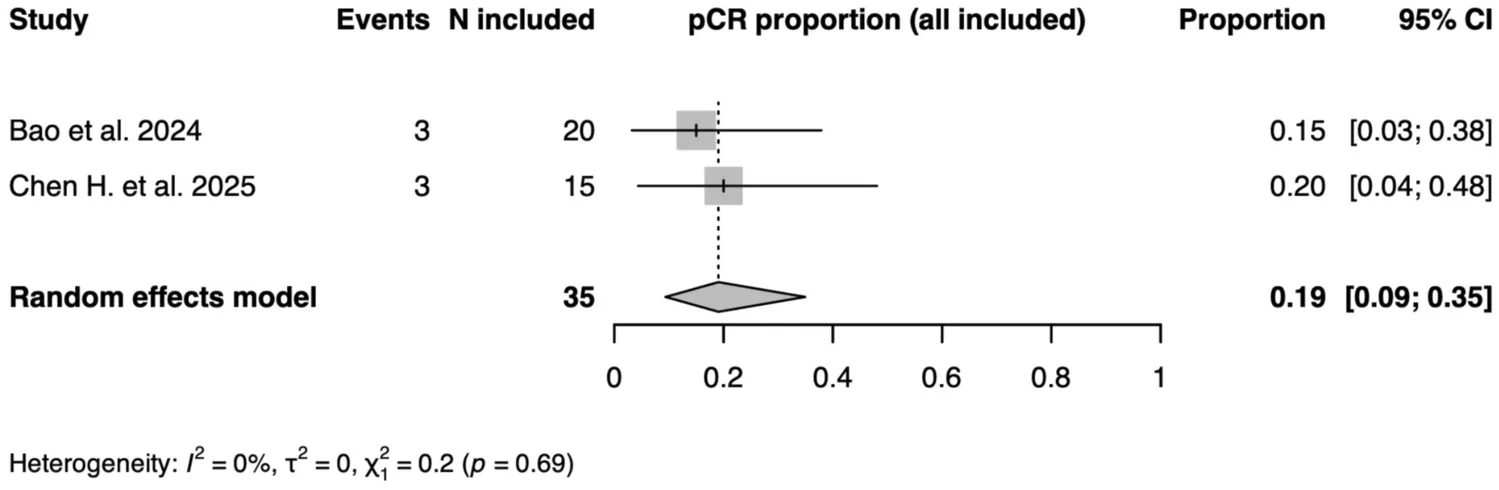
Forest plot of rates of pCR across included neoadjuvant UTUC studies

Two studies (N = 35) assessed the efficacy of DV with tislelizumab in patients with UTUC [32, 50]. The median age of included participants was 70 years. The estimated pCR rate was 19% (95% CI 9–35%, N = 35) and 23% (95% CI 11–43%, N = 26) among surgery-evaluable patients, without significant heterogeneity between trials (p = 0.69) (Fig. 3; Suppl. Figure 7). The rates of grade 3 or worse TRAEs were low (7.1% and 10.5%) without grade five events.

### Organ-sparing setting

#### Bladder-sparing

**Fig. 4:**
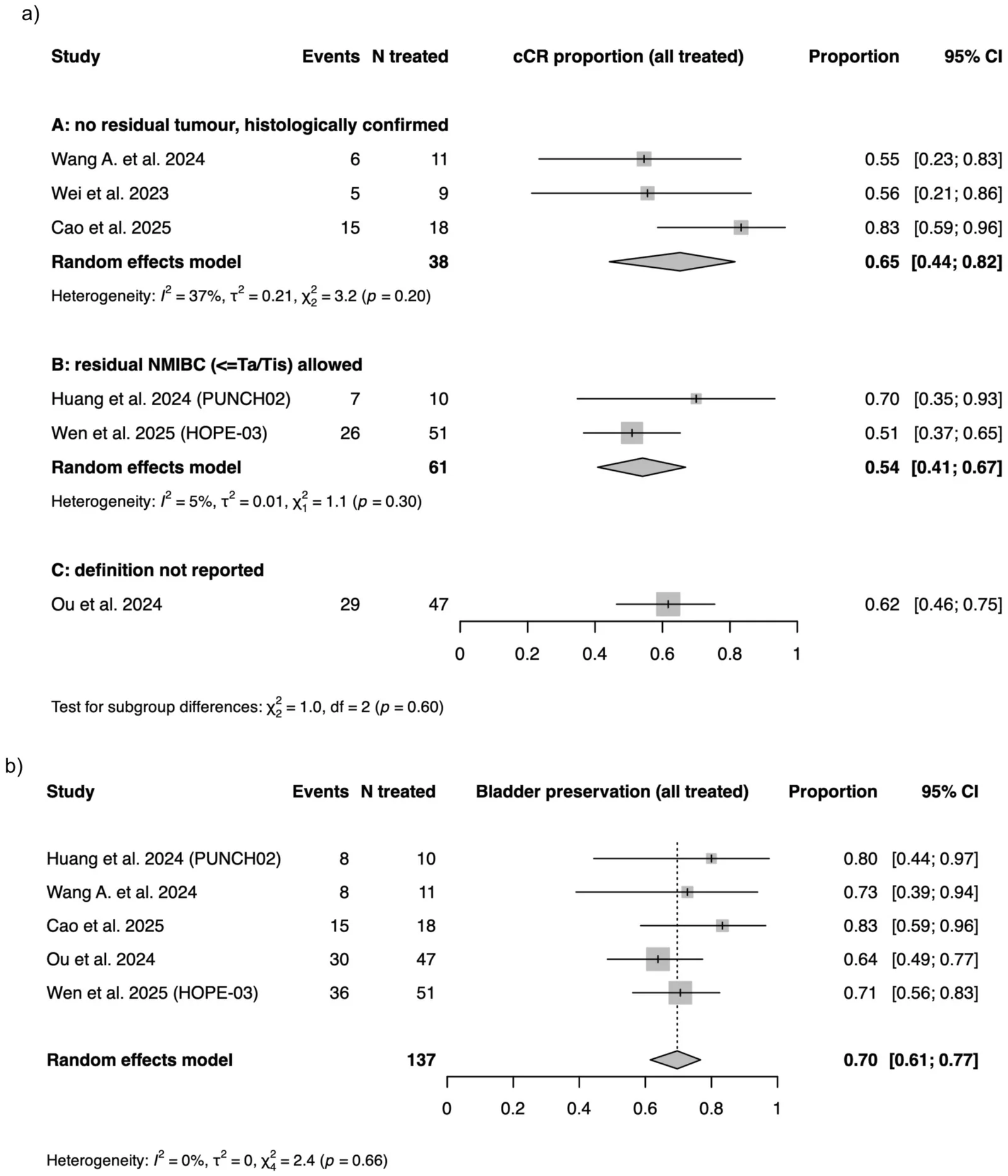
Forest plot of rates of **a** cCR and **b** bladder preservation among included bladder-sparing trials

Six trials (N = 146) [22, 31, 35, 37, 44, 45] evaluated a bladder-sparing strategy using DV and ICI following primary TURB. As cCR definitions varied substantially between studies (Table 2), results were stratified by response definition. In studies requiring complete absence of residual tumor [35, 44, 45], the pooled cCR rate was 65% (95% CI 44–82%, N = 38); in studies that allowed for residual non-muscle-invasive disease (≤ Ta/Tis) it was 54% (95% CI 41–67%, N = 61) [22, 31], and in one study without a reported definition, cCR was achieved in 29/47 (62%) patients (Fig. 4a) [37]. There was no significant difference between definition groups (p = 0.6).

**Table 2:** Studies with the HER2-targeted therapies in the organ-sparing setting

1st Author, Year	Study Design (Study Identifier)	Inclusion criteria	Publication type	cCR definition	N of Patients	N of Males	Median Age	Median FU	Treatment	HER2 expression				cCR [%]	Organ preserv [%]	Additional outcomes	TRAE [%]	TRAE G≥3 [%]	Most common TRAEs
										3+	2+	1+	0						
*Bladder-sparing*																			
Huang et al. 2024 [18]	Phase II (PUNCH02, ChiCTR2400080073)	HER2 (2+/3 +) cT2-4aN0M0	Abstract	normal urine cytology and MRI imaging, bladder biopsies ≤cTa	10	7	66.6	5.7	DV + Tislelizumab (4 cycles)	7	3			70	80			0	PNP, rash, alopecia
Wang A. et al. 2024 [19]	Retrospective	MIBC (T2N0M0-T4N2M0)	Article	radiologcial (RECIST 1.1) and cystoscopy visual assessment	11	5	67	19	DV+ (Toripalimab/ Tislelizumab) (at least 2 cycles)	3	5	3		54.5	72.7 at 16 mo	ORR = 81.8%	100	27.3	Decreased appetite, fatigue, rash, PNP, anemia, nausea
Wei et al. 2023 [20]	Retrospective	MIBC cT2-T4aN0-2M0	Article	MRI assessment (RECIST 1.1) with bladder biopsy/TURB-T	9	8	72	12	DV + Toripalimab/ Tislelizumab (at least 2 cycles)	4	3	1	1	55.6		ORR = 88.9% median rPFS = 12 mo	88.9	22.2	Decreased appetite, fatigue, rash
Cao et al. 2025	Retrospective	Previously untreted MIBC (cT2-T3N0-1M0) with HER2 expression of IHC ≥ 2+	Abstract	no residual tumor in re-TURBT, no tumor on imaging, negative urine cytology	18				DV + Toripalimab (6 cycles)	3	15			83.3	83.3	ORR = 94.4%			
Ou et al. 2024 [21]	Retrospective	MIBC (T2-4N0-1M0) HER2 (≥ 1 +)	Abstract	-	47	40	66	8	DV + Toripalimab	8	23	16		61.7	63.8	pDS=78.7% 1-yr BI-DFS=100%		25	Anemia, elevated ALT, fatigue
Wen et al. 2025 [22]	Phase Ib/II, SA (HOPE−03, ChiCTR2200060153)	MIBC (cT2-4bN0-3M0-1a) HER2 (≥ 1 +)	Article	cT0/Ta/Tis: no detectable tumour on chest/abdominal CT, bladder MRI and cystoscopy	51	44	70	12.8	DV + Tislelizumab	22	26	3		56.5	70.6	ORR = 86.95%	33.3**		PNP, erythema, elevated ALT and AST
*Bladder-sparing (TMT)*																			
Chen Y. et al. 2025 [23]	Retrospective	MIBC ineligible or refused radical cystectomy (RC) and received TURBT and synchrotron radiation (50–70 Gy) and/or RNU	Abstract	-	42***			38.1	RT + DV	42* only as positive						Relapse rate 35.7%	66.7	28.6	Elevated ALT and AST, pruritus, fatigue
Dahl et al. 2024 [24]	Phase I/II, non-randomized (NCT00238420)	Locally advanced MIBC, HER2 positive (IHC 2/3 +)	Article	Negative cystocsopy/umor biopsy^v^	20	18	79.5	2.3 yr	RT + paclitaxel + trastuzumab	20 (2/3 +)				72.2		2-yr OS=64.97% 3-yr OS = 56.15% 1-yr DFS = 63.95% 3-yr DFS = 25%		80	
Zhang L et al. 2025 [25]	Phase II (NCT05979740)	HER2 (IHC 2/3 +) T2-T4aN0M0	Article	Negative imaging, cystoscopy with biopsies, and urine cytology	6	5	69.5	12.3	RT + DV + Toripalimab (up to 12 cycles)	6 (2/3 +)				83.3			100	33.3	PNP, elevated ALT and AST, hyperlipidemia, rash, fatigue
*Kidney-sparing*																			
Ye et al. 2025 [26]	Phase II (WUTSUP-03)	localized UTUC patients (cT≤2N0M0) characterized by conditions such as solitary kidney, bilateral tumors, impending/already renal insufficiency and individual refusal/ineligibility for RNU	Abstract	-	34			18	DV + ICI (3–4 cycles)	3	24		7	73.53	91.18%	1-yr DFS = 67.65% 1-yr CFS = 91.18%		0	
Huang et al. 2025 [27]15–10-2026 12:56:00	Phase II (DISTINCT-I, NCT05912816)		Abstract	-	19			13	DV + Tislelizumab (2–4 cycles)	6	2	7	4	73.7		1-yr KI-EFS = 68.4%		0	

*MIBC* muscle-invasive bladder cancer, *UTUC* upper-tract urothelial carcinoma, *N* number, *FU* follow-up, *mo* month, *cCR* clinical complete response, *TRAE* treatment-related adverse events, *IHC* immunohistochemistry, *RC* + *PLND* radical cystectomy with pelvic lymph node dissection, *RNU* radical nephroureterectomy, *RT* radiotherapy, *DV* Disitamab vedotin, *ICI* immune-checkpoint inhibitor, *ORR* objective response rate, *rPFS* radiological progression-free survival, *pDS* pathological down-staging, *BI*-*DFS* bladder intact disease-free survival, *CFS* conversion-free survival, *KI*-*EFS* kidney-intact event-free survival, *PNP* peripheral sensory neuropathy

^**^7 patients experienced immune relates AEs, 10 patients experienced DV related AEs

^***^not stated how many UTUC patients

v—defined as complete response

The pooled bladder preservation rate was 70% (95% CI 61–77%, N = 137), with no evidence for significant heterogeneity (p = 0.66) (Fig. 4b). The most common TRAEs were PNP, alopecia and fatigue. In trial by Huang et al. there was no grade 3 or worse TRAE, in other trials it varied from 22 to 27%. No grade five events were reported [22, 37, 44, 45].

Additionally, three trials (N = 68) [23, 26, 34] assessed the role of trimodal therapy (TMT) in bladder-sparing setting with the use of HER2-targeted agents. Trial by Chen Y. et al. (N = 42) assessed efficacy of DV combined with radiotherapy [34], trial by Dahl et al. (N = 20) the combined treatment with paclitaxel and trastuzumab [23], and trial by Zhang L. et al. (N = 6) DV with toripalimab [26]. Because response definitions differed (cystoscopy and/or biopsy in Dahl et al.; imaging, cystoscopy with biopsy and cytology in Zhang L. et al.), results were not pooled: complete response was achieved in 13/20 (65%) and 5/6 (83%) patients, respectively. Importantly in trial by Dahl et al. the rate of grade ≥3 TRAEs was 80%, while in two trials using DV it was around 30% (predominantly PNP and elevated liver enzymes) [26, 34].

#### Kidney-sparing

HER2-targeted therapy has been also evaluated as a kidney-sparing strategy in patients with UTUC with DV+ICI. In WUTSUP-03, 1-year disease-free survival was 67.65% and 91.18% of patients maintained their kidneys during the first year after treatment [20]. In DISTINCT-I, kidney preservation at 1-year was 68.4% [21]. Neither study reported criteria for cCR; cCR was achieved in 25/34 (74%) and 14/20 (70%) patients, respectively. None of the studies reported TRAEs of grade 3 or worse.

### Adjuvant setting

#### Bladder cancer

Two trials assessed the efficacy of HER2-targeted agents as adjuvant treatment in patients with MIBC [25, 46] (Table 3). NeuACT trial assessed the role of DN24-02 autologous cellular immunotherapy in comparison with standard of care (SOC) surveillance in 142 patients with high-risk MIBC. There was no significant difference in OS (HR 0.96; p =0.87) or recurrence-free survival (RFS) (HR 0.99; p = 0.97), and the trial was prematurely terminated for futility [25].

The second trial by Xu et al. assessed the role of adjuvant DV in patients with tumors confined to bladder or ureter after definitive treatment, in the cohort of 32 patients the recurrence rate in DV group was significantly lower compared to chemotherapy group (9.4% vs. 20.2%, p = 0.007). The rate of adverse events was also lower in the DV group (12.5% vs. 20.2%, p = 0.022) [46].

#### Upper-tract urothelial cancer

The role of adjuvant therapy in UTUC was evaluated in three trials testing DV [24, 33, 47] (Table 3). In the first study by Liu S. et al. in patients after radical surgery without neoadjuvant therapy DV was combined with toripalimab, resulting in 1-year disease-free survival (DFS) of 90%, with 20% of patients experiencing serious TRAEs [33]. In the second trial by Tao et al. [24] in patients who were cisplatin-unfit and high-risk after nephroureterectomy DV was combined with radiotherapy, with no recurrences within six months after treatment and 6.7% rate of serious TRAEs. Finally, in the study by Wang et al. [47] in patients after nephroureterectomy DV was combined with PD-1 inhibitor, achieving 15-month DFS of 76.2% with 42.9% of patients experiencing TRAEs.

## Discussion

In this systematic review and meta-analysis, HER2-directed regimens, predominantly DV-containing combinations, showed promising activity across perioperative strategies in patients with UC. In the neoadjuvant setting in BC, the pooled pCR rate was 48%, with pDS reaching 74%. Evidence was insufficient to establish an association between HER2 expression and response. In organ-preserving cohorts, reported responses were encouraging for both bladder- and kidney-sparing regimens; however, follow-up periods were short and reported outcomes, especially cCR varied in their definitions. In the adjuvant setting, outcomes were heterogeneous and the available data did not permit formal meta-analysis. DN24-02 failed to improve survival; yet the emerging data on DV seem promising, and larger cohorts with longer follow-up are needed for definitive conclusions.

While majority of the included studies were available only as conference abstracts and DV was combined with various ICIs, eligibility criteria and analysis populations differed across studies. Importantly, pooled estimates reflect DV-containing regimens and do not isolate effect of HER2 targeting. In the neoadjuvant setting, patients with treatment-naïve, locally-advanced disease were included. Similarly, in organ-sparing cohorts, disease was predominantly locally advanced, apart from two trials including patients ineligible or who refused operation [20, 34].

In the neoadjuvant setting, the pooled pCR rate of 48% achieved with DV-containing regimens is promising and worth testing in randomized trials, as those results are comparable with response achieved in previous trials with single agent ICIs (ABACUS, NABUCCO or PURE-01) [54–56], where the pCR rates ranged from 31 to 46%. When compared with other ADCs, combination EV and pembrolizumab in KEYNOTE−905 achieved pCR of 57% in the cisplatin-ineligible patients, while in our analysis pCR in the cisplatin-unfit cohort it was 38% [57]. In trial KEYNOTE-B15 in cisplatin-eligible population a pCR rate was 55.8% [58]. Yet, these results should be interpreted cautiously as only three studies assessed defined cisplatin-ineligible cohort [27, 36, 52]. Other included trials did not specify if they included cisplatin-unfit patients.

Our analysis showed that currently there is insufficient evidence to establish association between HER2 expression and response. It should not be interpreted as evidence that HER2 expression does not influence response, but there is evidence showing HER2-directed ADCs remain active in tumors with low HER2 expression in breast cancer [59]. It could potentially be explained by the pharmacology of ADCs—as the, payload release and bystander effect may reduce dependence on high target expression, but sample size was too small to draw definitive conclusions [60, 61]. Importantly, majority of included studies used standard immunohistochemistry (IHC), there is no centralized protocol for HER2-expression assessment in UC [62]. Staining was highly center-dependent with various antibodies, cut-off values, scoring systems and source of tissue across studies. In addition, only a minority of studies reported outcomes stratified by HER2 expression, limiting statistical power.

A further constraint is that evidence comprises mainly of surrogate endpoint, with most studies reporting pCR (neoadjuvant) and cCR (organ-sparing) and mature survival data are limited. Although pCR has been proven to be an early efficacy signal [63], cCR is not a reliable outcome, confirmative for true disease eradication [64]. Also its definition varied across studies (Table 2) some studies required complete absence of residual tumor, whereas others permitted residual non-muscle-invasive disease; for example in PUNCH02, cCR was defined as normal urine cytology and MRI imaging with bladder biopsies ≤cTa [22], and only in trials by Cao et al., Dahl et al. and Zhang et al. it was confirmed by biopsy [23, 26, 35]. While some of the included studies were supported with the liquid-biopsies analyses, this approach needs further validation in the prospective randomized trials. Moreover, the short follow-up in most organ-sparing studies is insufficient to mitigate the risk serious AEs associated with the treatment.

**Table 3:** Studies with the HER2-targeted therapies in the adjuvant setting

1st Author, Year	Study Design (Study Identifier)	Inclusion criteria	Publication type	N of Patients	N of Males	Median Age	Median FU	Treatment Drug	HER2 expression				Additional outcomes	TRAE [%]	TRAE G≥3 [%]	Most common TRAEs
									3+	2+	1+	0				
*MIBC*																
Bajorin et al. 2016 [27]	Phase II, randomized (NeuACT, NCT01353222)	High-risk MIBC post-RC HER2 positive (IHC ≥1)	Abstract	142 (both arms)			13.2	DN24-02 (3 infusions)					Median OS = 37 mo Median DRFS = 11.9 mo			
Xu et al. 2023 [28]	Retrospective	The tumors in these patients were confined to the bladder or ureter. Surgical treatment options included TURB, RC, RNU; depending on the tumor location and size HER-2 positivite (IHC ≥1)	Article	32	21	69.2 (mean)		DV (3 months)	5	16	11	0	Recurrence rate = 9.4%	21.9	12.5	Anemia, PNP, nausea, decreased appetite
*UTUC*																
Liu S. et al. 2025 [29]	Phase II, SA (NCT05917158)	histologically confirmed HER2 IHC 2+/3+ UTUC after radical surgery and were staged as T2-4NanyM0 or TanyN1-2M0, with no prior neoadjuvant therapy	Abstract	45	35	68	12.2	DV + Toripalimab (6 cycles + toripalimab up to 1 year)	9	36			1-y DFS=90%	100	20	Nausea, pruritus, rash, hypothyroidism
Tao et al. 2025 [30]	Phase II, open label, non-randomized (NCT06210490)	cisplatin-intolerant pts with HER2- overexpressing (Immunohistochemistry, IHC 2+, 3 +) UTUC after RNU staged as pT2N0M0 with high-grade/multifocal disease/positive surgical margin, pT3-4 N0 M0 or pTany N1-2 M0	Abstract	15			6	DV + RT (DV for 6 months)					6-mo DFS = 100%		6.7	Abdominal pain, rash, leukopenia, elevated Alt and AST
Wang et al. 2025 [31]	Retrospective	Locally advanced HER2 positive (IHC 2/3 +).UTUC post RNU	Abstract	21	13	68	15	DV + PD-1 inhibitor (at least 5 cycles + PD-1 inhibitor up to 1 year)	15	6			15-mo DFS = 76.2%	42.9		

*MIBC* muscle-invasive bladder cancer, *UTUC* upper-tract urothelial carcinoma, *N* number, *FU* follow-up, *mo* month, *cCR* clinical complete response, *TRAE* treatment-related adverse events, *IHC* immunohistochemistry, *TURB* transurethral resection of bladder tumor, *RC* radical cystectomy, *RNU* radical nephroureterectomy, *DV* Disitamab vedotin, *ICI* immune-checkpoint inhibitor, *RT* radiotherapy, *OS* overall survival, *DRFS* disease -free survival

See References [19, 20, 22–34, 36–46, 48, 50–52]

Primary limitations of this study include the predominance of single-arm trials with small sample sizes, many of which were published as conference abstracts, and Chinese databases were searched through October 14, 2025. Second, there were substantial differences in the number of treatment cycles across included trials. Third, in several studies, BC and UTUC were analyzed together, which precludes the exact characteristic of HER2 expression and treatment response within each cohort. Fourth, majority of these studies were conducted in China and used DV, currently not approved outside China, which was often combined with ICIs such as tislelizumab and toripalimab which are also not available outside China. It limits the generalizability of the findings and further validation in western population is needed. The difficulties in introducing anti-HER2 therapies in the world-wide practice are also underscored by other authors and remain one of the biggest challenge in current uro-oncology practice [65]. So far there is only few ongoing international trial assessing efficacy of DV in UC (NCT04879329 and NCT05911295) [66, 67]. Additionally, proportion of treated patients undergoing surgery varied across studies; partial overlap between cohorts from the same centers could not be excluded. Finally, as ROBINS-I was developed for comparative non-randomized studies, its application to single-arm data required adaptation, and bias due to confounding could not be assessed.

## Conclusion

Neoadjuvant DV-containing regimens show promising activity in patients with bladder cancer. Current evidence is insufficient to establish an association between HER2 expression and response. Emerging data indicate a possible role in the bladder-sparing setting with low rates of toxicity and no G5 safety signals. However, current evidence is based on single-arm studies with surrogate endpoints, limited follow-up and heterogeneous cCR definitions. Prospective, comparative trials with standardized HER2 assessment, survival follow-up and global validation, preferably already integrating molecular response biomarker, are needed to comprehensively define role of HER2-targeted ADCs in perioperative UC setting.

## Supplementary Information

Below is the link to the electronic supplementary material.

Supplementary file1 (DOCX 270 KB)

Supplementary file2 (DOCX 264 KB)

Supplementary file3 (DOCX 15 KB)

Supplementary file4 (DOCX 32 KB)

Supplementary file5 (DOCX 83 KB)

Supplementary file6 (TIFF 115910 KB)

Supplementary file7 (PDF 4199 KB)

## Data Availability

All data analyzed during this study are derived from previously published studies, which are cited in the manuscript. The extracted data supporting the findings of this meta-analysis are available within the article and its supplementary information files.
