# Supplementary material for: Efficacy of HER2-targeted therapies in localized urothelial carcinoma: a systematic review and meta-analysis
Source: World J Urol. 2026 Nov 14;44(1):810. doi: 10.1007/s00345-026-06868-2 (PMC13769737; doi:10.1007/s00345-026-06868-2)
Supplement: Supplementary file 1 [file 345_2026_6868_MOESM7_ESM.pdf]

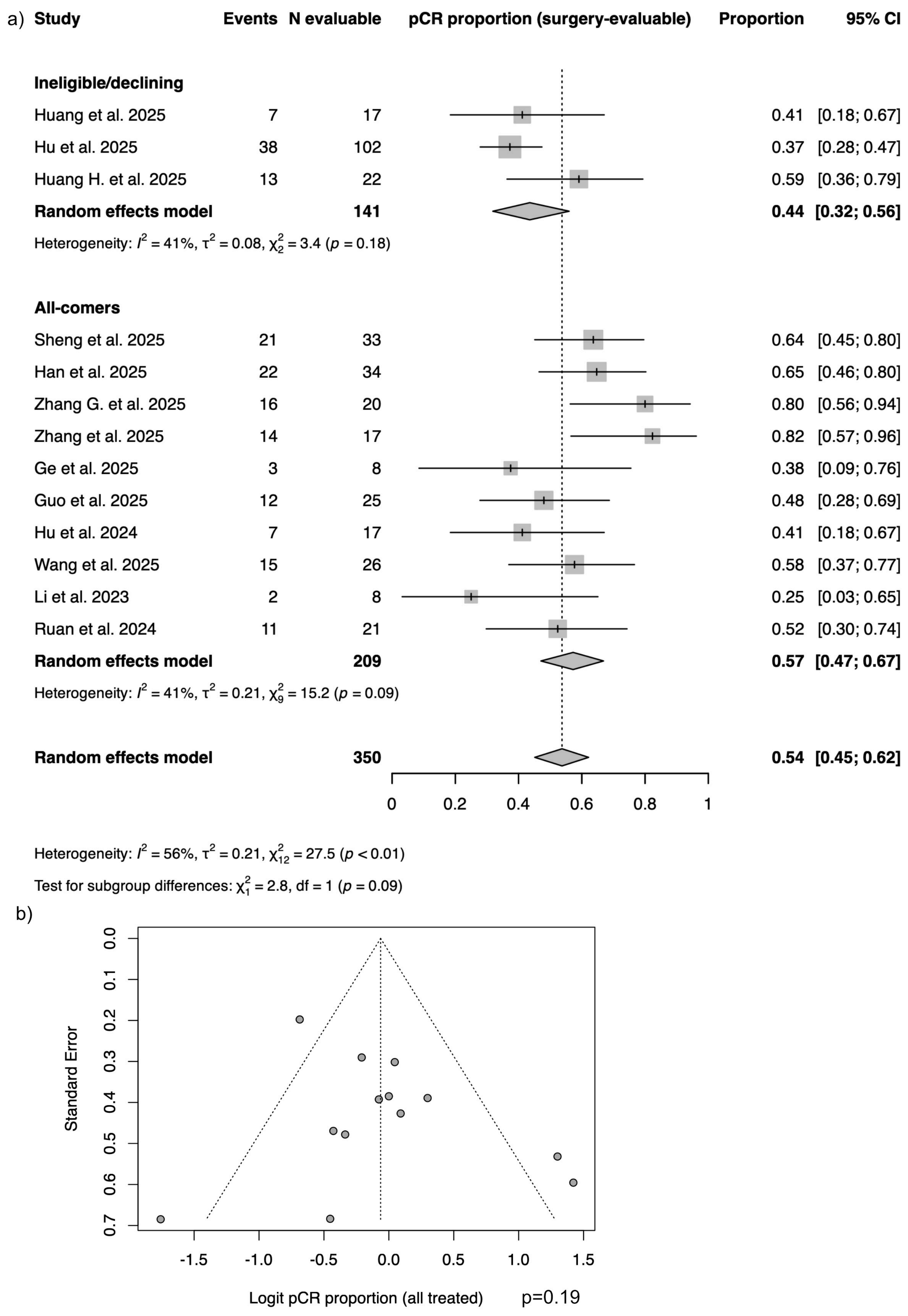
a)
Study | Events | N evaluable | pCR proportion (surgery-evaluable) | Proportion | 95% CI
Ineligible/declining
Huang et al. 2025 7 17 0.41 [0.18; 0.67]
Hu et al. 2025 38 102 0.37 [0.28; 0.47]
Huang H. et al. 2025 13 22 0.59 [0.36; 0.79]
Random effects model 141 0.44 [0.32; 0.56]
Heterogeneity: $I^2 = 41\%$, $\tau^2 = 0.08$, $\chi^2_2 = 3.4$ ($p = 0.18$)
All-comers
Sheng et al. 2025 21 33 0.64 [0.45; 0.80]
Han et al. 2025 22 34 0.65 [0.46; 0.80]
Zhang G. et al. 2025 16 20 0.80 [0.56; 0.94]
Zhang et al. 2025 14 17 0.82 [0.57; 0.96]
Ge et al. 2025 3 8 0.38 [0.09; 0.76]
Guo et al. 2025 12 25 0.48 [0.28; 0.69]
Hu et al. 2024 7 17 0.41 [0.18; 0.67]
Wang et al. 2025 15 26 0.58 [0.37; 0.77]
Li et al. 2023 2 8 0.25 [0.03; 0.65]
Ruan et al. 2024 11 21 0.52 [0.30; 0.74]
Random effects model 209 0.57 [0.47; 0.67]
Heterogeneity: $I^2 = 41\%$, $\tau^2 = 0.21$, $\chi^2_9 = 15.2$ ($p = 0.09$)
Random effects model 350 0.54 [0.45; 0.62]
0 0.2 0.4 0.6 0.8 1
Heterogeneity: $I^2 = 56\%$, $\tau^2 = 0.21$, $\chi^2_{12} = 27.5$ ($p < 0.01$)
Test for subgroup differences: $\chi^2_1 = 2.8$, df = 1 ($p = 0.09$)
b)
Standard Error
0.0 0.1 0.2 0.3 0.4 0.5 0.6 0.7
-1.5 -1.0 -0.5 0.0 0.5 1.0 1.5
Logit pCR proportion (all treated)
p=0.19

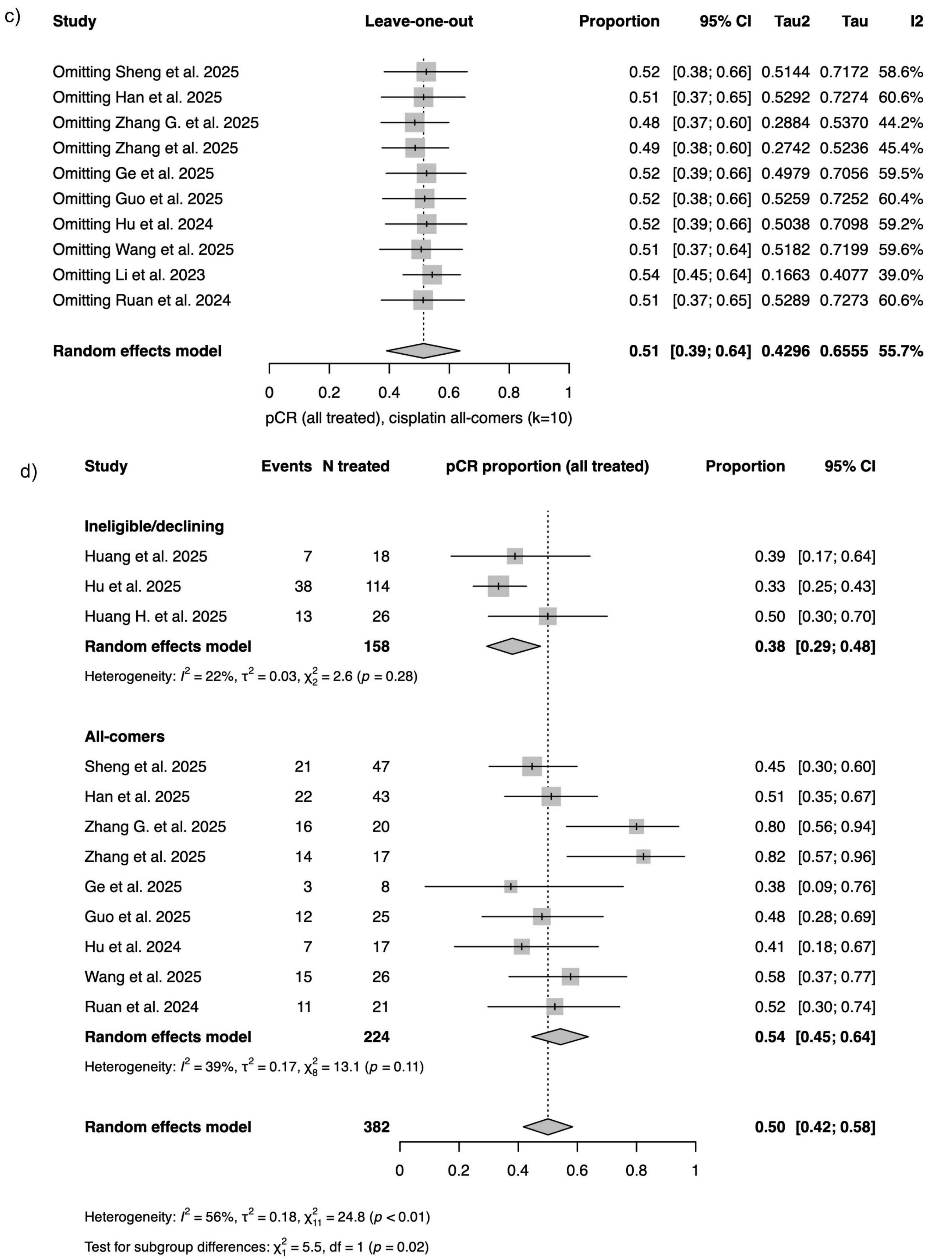

Suppl. Figure 1.a) pCR rates among surgery-evaluable patients by cisplatin eligibility;
b) funnel plot (Egger's test p=0.19), all treated; c) leave-one-out analysis in the all-comers cohort;
d) pCR rates by cisplatin eligibility after excluding Li et al. 2023 (all treated)

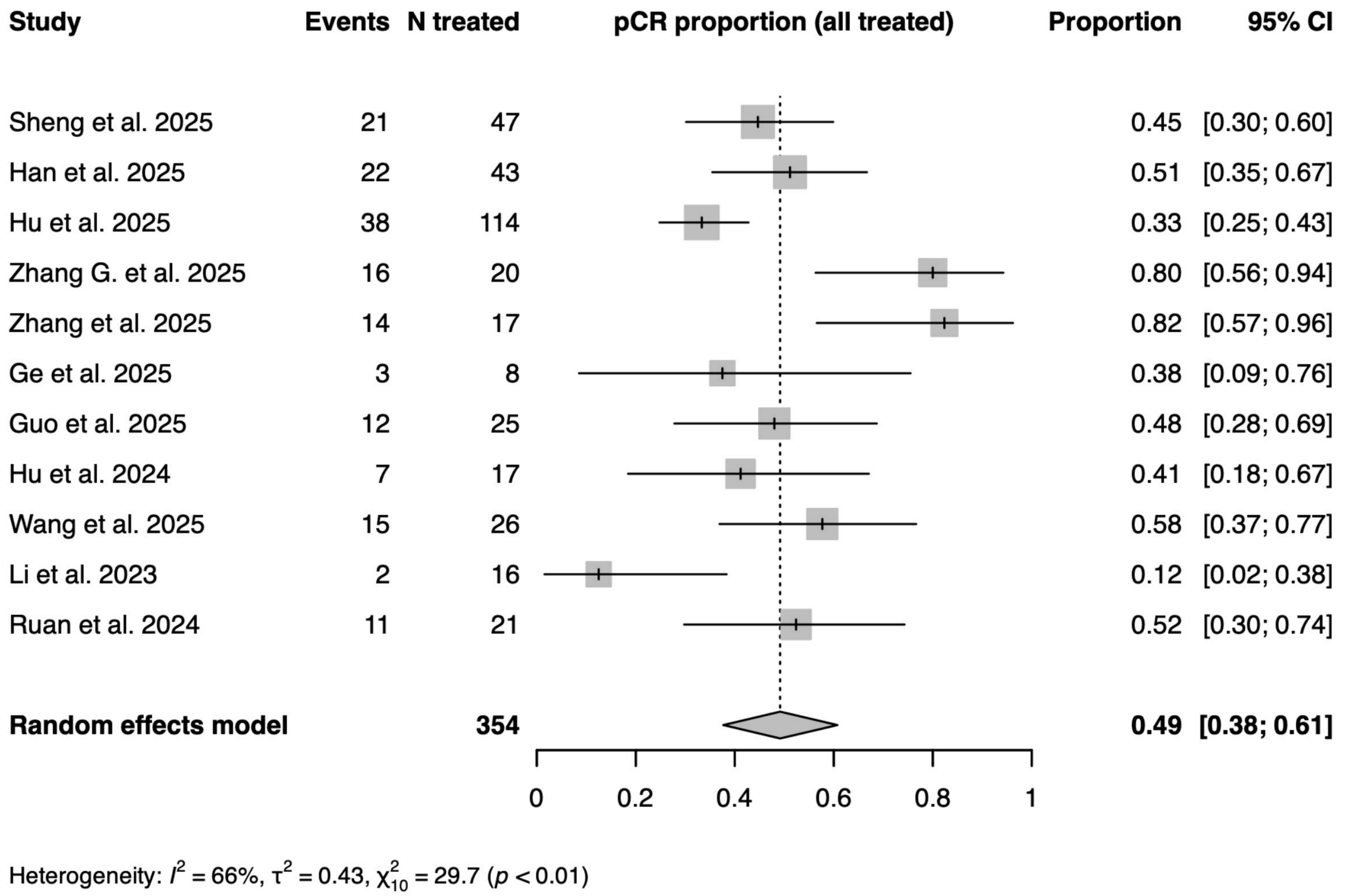

Supplementary Figure 2. Forest plot of rates of pCR across included neoadjuvant bladder cancer studies using only disitamab vedotin combined with immune checkpoint inhibitors

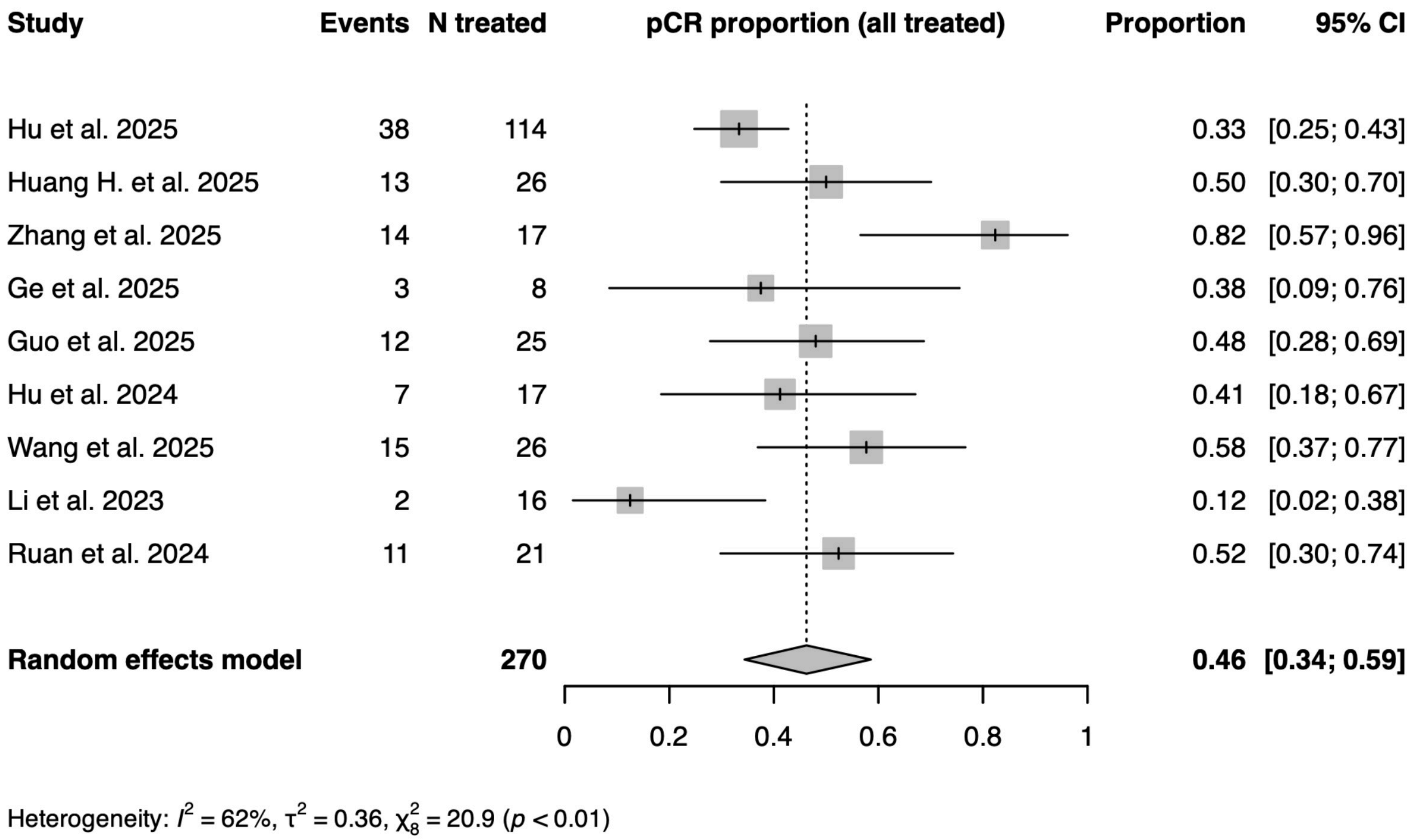

Supplementary Figure 3. Forest plot of pCR rates across studies published as full-text articles

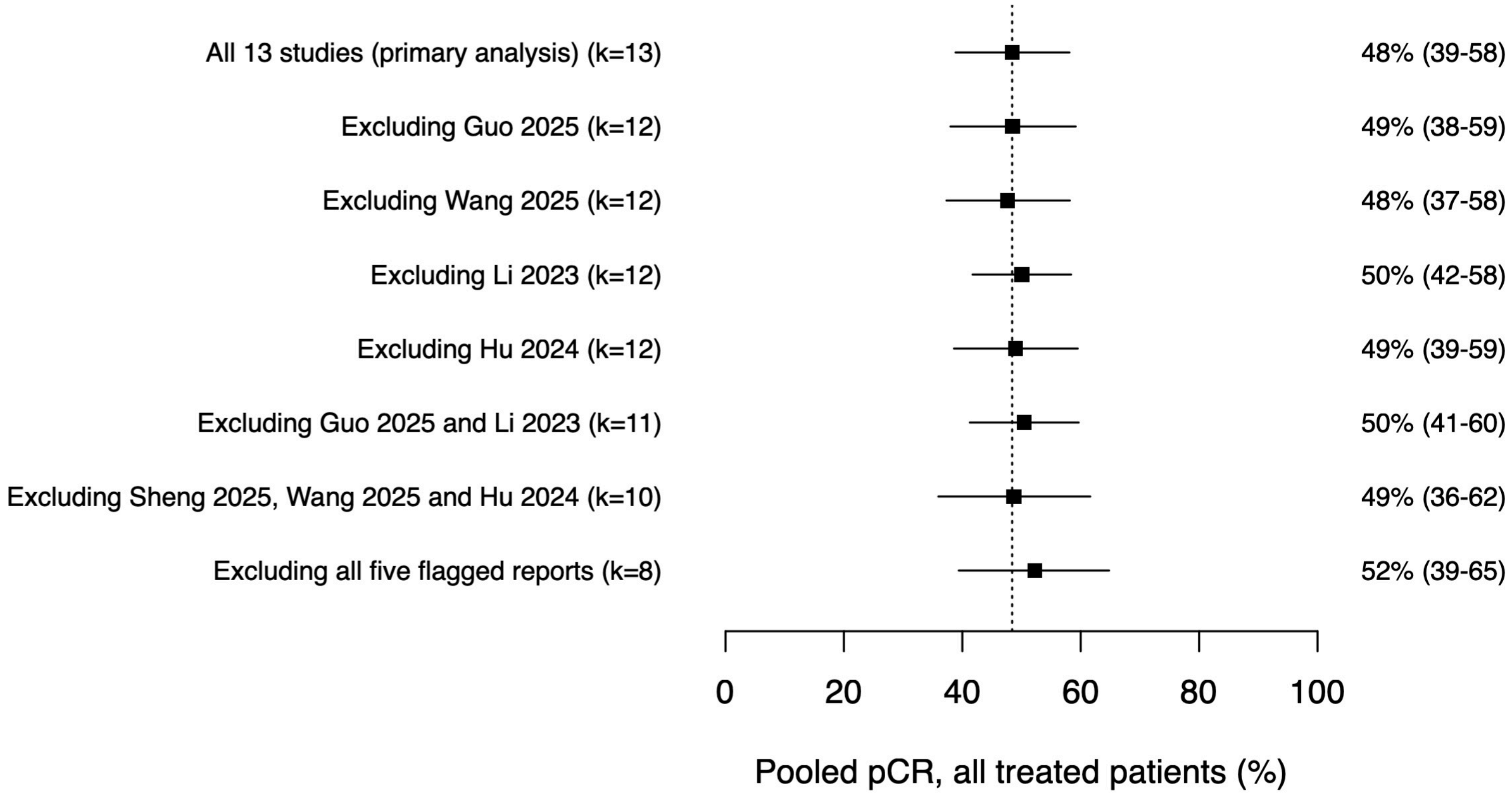

Supplementary Figure 4. Plot of pooled pCR excluding potentially overlapping studies

a)

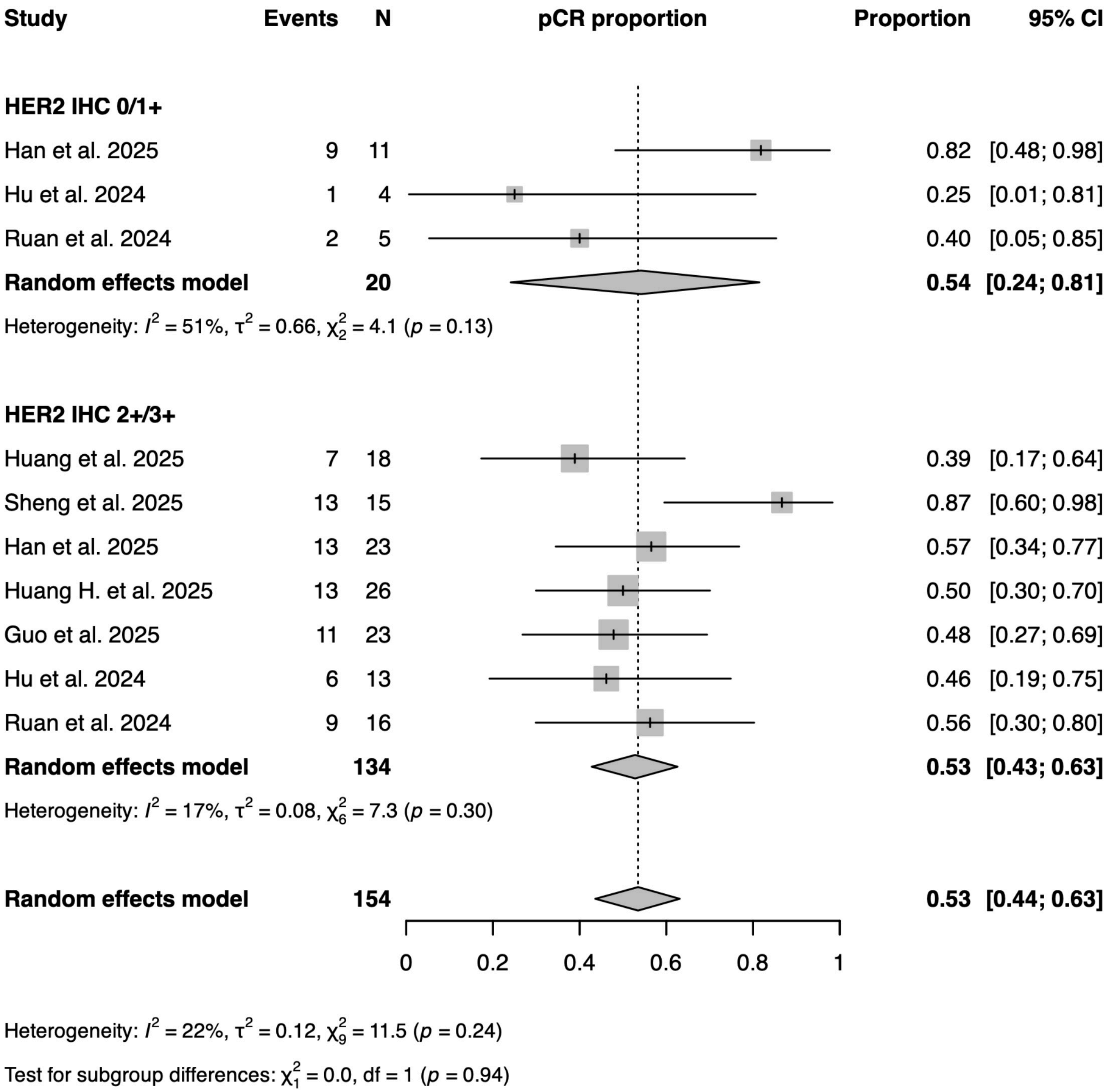

b)

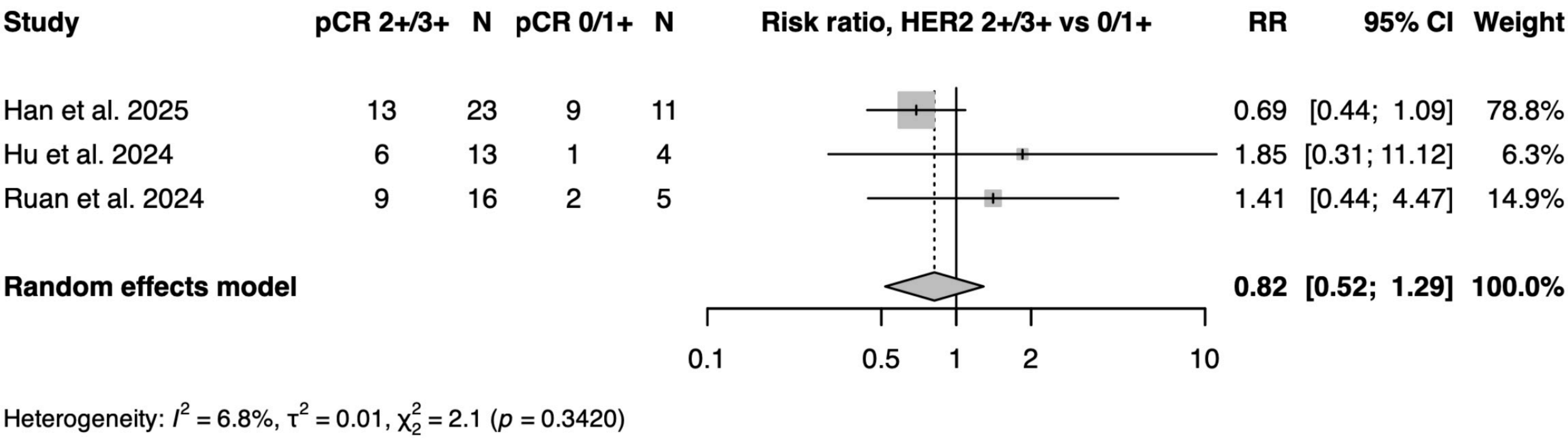

Supplementary Figure 5. The estimated rate of pCR across tumors with HER2 expression a) 0/1+ and 2/3+ b) comparison of response between HER2 2/3+ and HER2 0/1

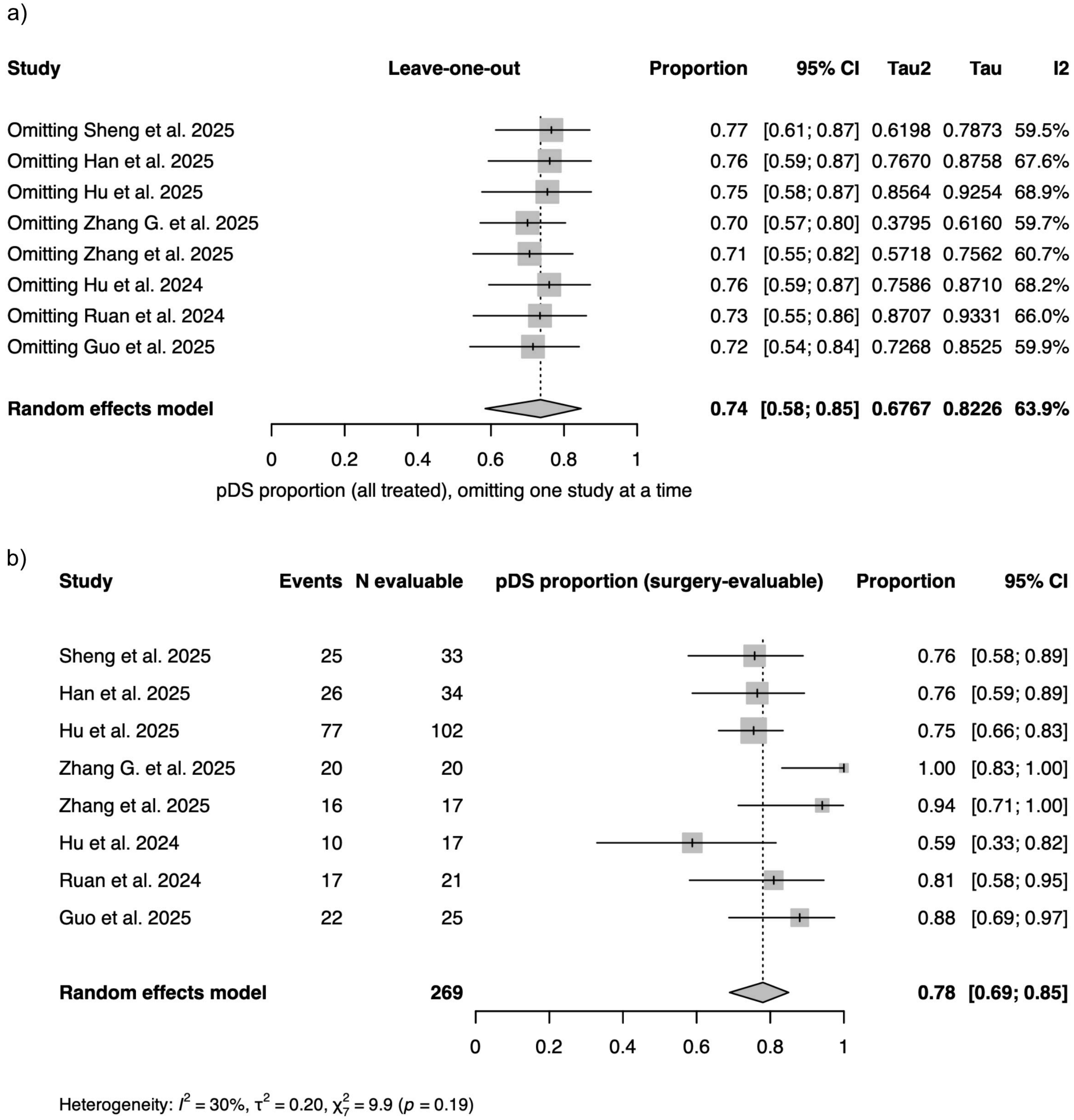

Supplementary Figure 6. (a) a) Leave-one-out sensitivity analysis pathological downstaging b) Forest plot of rates of pathological down-staging across included neoadjuvant bladder cancer studies (surgery-evaluable)

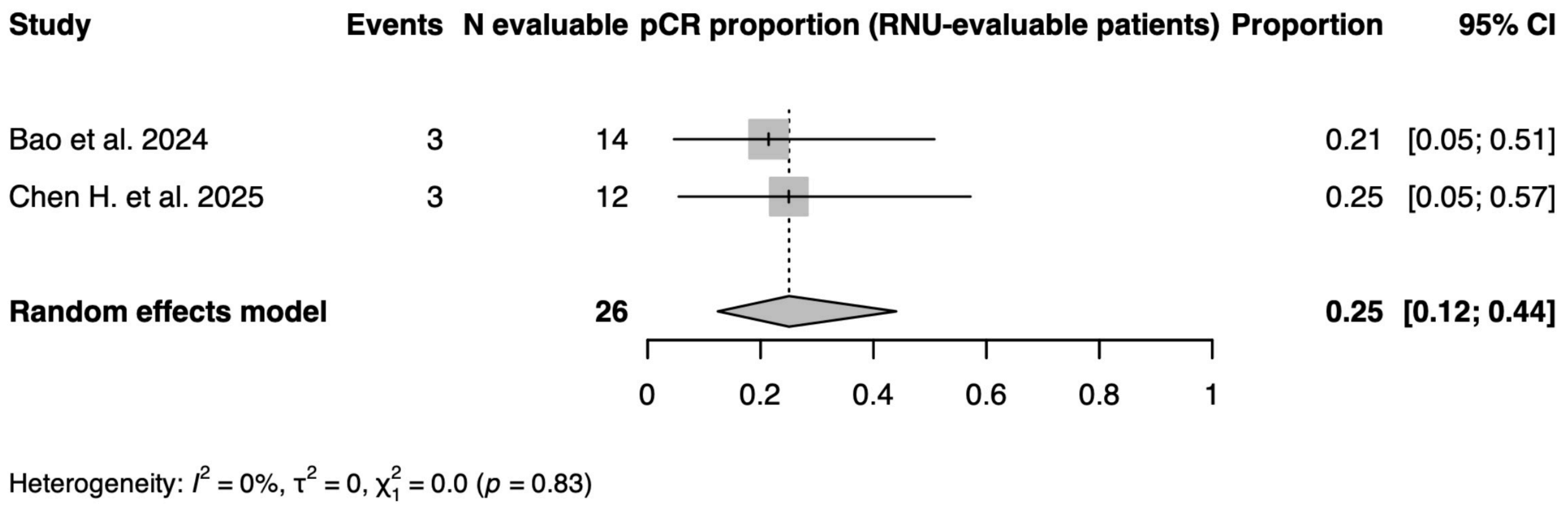

Supplementary Figure 7. Forest plot of rates of pCR across included neoadjuvant UTUC studies (surgery evaluable)
