# Supplementary material for: Efficacy of HER2-targeted therapies in localized urothelial carcinoma: a systematic review and meta-analysis
Source: World J Urol. 2026 Nov 14;44(1):810. doi: 10.1007/s00345-026-06868-2 (PMC13769737; doi:10.1007/s00345-026-06868-2)
Supplement: Supplementary file 4 [file 345_2026_6868_MOESM3_ESM.docx]

Supplementary Table: Study design according to the Population, Intervention, Control, Outcome, Study Design (PICOS) method.

| Population (P) | Patients with localized urothelial carcinoma (UC) |
| --- | --- |
| Intervention (I) | Treatment with HER-2 directed therapy |
| Control (C) | - |
| Outcome (O) | Pathological complete response (pCR), clinical complete response (cCR), pathological downstaging (pDS), objective response rate (ORR), bladder preservation rate and safety of treatment |
| Study design (S) | Single-arm trials, retrospective studies |
