# Supplementary material for: Efficacy of HER2-targeted therapies in localized urothelial carcinoma: a systematic review and meta-analysis
Source: World J Urol. 2026 Nov 14;44(1):810. doi: 10.1007/s00345-026-06868-2 (PMC13769737; doi:10.1007/s00345-026-06868-2)
Supplement: Supplementary file 5 [file 345_2026_6868_MOESM4_ESM.docx]

Supplementary File x - Search strategy:

Database(s): MEDLINE, PubMed Central, and Bookshelf (via PubMed platform)

Executed on 16/09/2025. No date or language filters were used.

| # | Search string | Results |
| --- | --- | --- |
| 1 | "ErbB Receptors"[MeSH] OR "HER2*" [Title/Abstract] OR "HER-2*" [Title/Abstract] OR "HER/NEU" [Title/Abstract] OR "epidermal growth factor receptor*"[Title/Abstract] OR EGFR2*[TIAB] OR "ERBB2*" [Title/Abstract] OR "ERBB-2*" [Title/Abstract] OR "C-ERBB2*" [Title/Abstract] OR "C-ERBB-2*" [Title/Abstract] OR "ERB-B2*" [Title/Abstract] OR "ERB-B-2*" [Title/Abstract] OR "C-ERB-B2*" [Title/Abstract] OR "C-ERB-B-2*" [Title/Abstract] OR "MLN19" [Title/Abstract] OR "MLN-19" [Title/Abstract] OR "p185erbB2" [Title/Abstract] OR "CD340*" [Title/Abstract] OR "CD-340*" [Title/Abstract] OR "NEU" [Title/Abstract] OR "NGL"[Title/Abstract] | 150,826 |
| 2 | "anti-HER*"[Title/Abstract] OR "antiHER*"[Title/Abstract] OR "Trastuzumab*"[Title/Abstract] OR "Herceptin"[Title/Abstract] OR "R-597"[Title/Abstract] OR "MYL-1401O"[Title/Abstract] OR "CT-P6"[Title/Abstract] OR "SB3"[Title/Abstract] OR "ABP-980" [Title/Abstract] OR "PF-05280014" [Title/Abstract] OR "HLX02" [Title/Abstract] OR "EG12014" [Title/Abstract] OR "Herwenda" [Title/Abstract] OR "Hercessi" [Title/Abstract] OR "Zercepac" [Title/Abstract] OR "Trazimera" [Title/Abstract] OR "Kanjinti" [Title/Abstract] OR "Ontruzant" [Title/Abstract] OR "Credima" [Title/Abstract] OR "Herzuma" [Title/Abstract] OR "Ogivri" [Title/Abstract] OR "Canmab" [Title/Abstract] OR "Hertraz" [Title/Abstract] OR "Pertuzumab" [Title/Abstract] OR "Perjeta" [Title/Abstract] OR "Ado-trastuzumab" [Title/Abstract] OR "Lapatinib" [Title/Abstract] OR "Kadcyla" [Title/Abstract] OR "Tykerb" [Title/Abstract] OR "Neratinib" [Title/Abstract] OR "Nerlynx" [Title/Abstract] OR "Tucatinib" [Title/Abstract] OR "Tukysa" [Title/Abstract] OR "Fam-trastuzumab deruxtecan" [Title/Abstract] OR "Enhertu" [Title/Abstract] OR "Margetuximab" [Title/Abstract] OR "Margenza" [Title/Abstract] OR "Zanidatamab" [Title/Abstract] OR "Ziihera" [Title/Abstract] OR "2C4" [Title/Abstract] OR "Herclon" [Title/Abstract] OR "MM-111" [Title/Abstract] OR "Tyverb" [Title/Abstract] OR "T-DM1" [Title/Abstract] OR "HKI-272" [Title/Abstract] OR "neratinib" [Title/Abstract] OR "Disitamab vedotin" [Title/Abstract] | 24,827 |
| 3 | "urinary bladder neoplasms"[MeSH Terms] OR ((bladder[Title/Abstract] OR urothelial[Title/Abstract] OR "upper tract"[Title/Abstract]) AND (cancer*[Title/Abstract] OR carcinoma*[Title/Abstract] OR neoplasm*[TIAB] OR tumor*[TIAB] OR tumour*[TIAB])) | 115,794 |
| 4 | patient*[TIAB] AND (treatment[TIAB] OR therapy[TIAB] OR monotherapy[TIAB] OR adjuvant[TIAB] OR neoadjuvant[TIAB] OR enrolled[TIAB] OR received[TIAB] OR treated[TIAB] OR assigned[TIAB] OR administered[TIAB]) | 4,491,590 |
| 5 | (#1 OR #2) AND #3 AND #4 | 625 |

Database(s): Embase via Embase.com

Executed on 13/2/2025. No date or language filters were used.

| # | Embase | # hits |
| --- | --- | --- |
| #1 | ('erbb receptors'/exp OR 'her2*':ti,ab,kw OR 'her-2*':ti,ab,kw OR 'her/neu':ti,ab,kw OR 'epidermal growth factor receptor*':ti,ab,kw OR egfr2*:ti,ab,kw OR 'erbb2*':ti,ab,kw OR 'erbb-2*':ti,ab,kw OR 'c-erbb2*':ti,ab,kw OR 'c-erbb-2*':ti,ab,kw OR 'erb-b2*':ti,ab,kw OR 'erb-b-2*':ti,ab,kw OR 'c-erb-b2*':ti,ab,kw OR 'c-erb-b-2*':ti,ab,kw OR 'mln19':ti,ab,kw OR 'mln-19':ti,ab,kw OR 'p185erbb2':ti,ab,kw OR 'cd340*':ti,ab,kw OR 'cd-340*':ti,ab,kw OR 'neu':ti,ab,kw OR 'ngl':ti,ab,kw) AND [embase]/lim | 233,849 |
| #2 | ('anti-her*':ti,ab,kw OR 'antiher*':ti,ab,kw OR 'trastuzumab*':ti,ab,kw OR 'herceptin':ti,ab,kw OR 'r-597':ti,ab,kw OR 'myl-1401o':ti,ab,kw OR 'ct-p6':ti,ab,kw OR 'sb3':ti,ab,kw OR 'abp-980':ti,ab,kw OR 'pf-05280014':ti,ab,kw OR 'hlx02':ti,ab,kw OR 'eg12014':ti,ab,kw OR 'herwenda':ti,ab,kw OR 'hercessi':ti,ab,kw OR 'zercepac':ti,ab,kw OR 'trazimera':ti,ab,kw OR 'kanjinti':ti,ab,kw OR 'ontruzant':ti,ab,kw OR 'credima':ti,ab,kw OR 'herzuma':ti,ab,kw OR 'ogivri':ti,ab,kw OR 'canmab':ti,ab,kw OR 'hertraz':ti,ab,kw OR 'ado-trastuzumab':ti,ab,kw OR 'lapatinib':ti,ab,kw OR 'kadcyla':ti,ab,kw OR 'tykerb':ti,ab,kw OR 'nerlynx':ti,ab,kw OR 'tucatinib':ti,ab,kw OR 'tukysa':ti,ab,kw OR 'fam-trastuzumab deruxtecan':ti,ab,kw OR 'enhertu':ti,ab,kw OR 'margetuximab':ti,ab,kw OR 'margenza':ti,ab,kw OR 'zanidatamab':ti,ab,kw OR 'ziihera':ti,ab,kw OR 'pertuzumab':ti,ab,kw OR 'perjeta':ti,ab,kw OR '2c4':ti,ab,kw OR 'herclon':ti,ab,kw OR 'mm-111':ti,ab,kw OR 'tyverb':ti,ab,kw OR 't-dm1':ti,ab,kw OR 'hki-272':ti,ab,kw OR 'neratinib':ti,ab,kw OR 'disitamab vedotin':ti,ab,kw) AND [embase]/lim | 40,353 |
| #3 | ('urinary bladder neoplasms'/exp OR ((bladder:ti,ab,kw OR urothelial:ti,ab,kw OR 'upper tract':ti,ab,kw) AND (cancer*:ti,ab,kw OR carcinoma*:ti,ab,kw OR neoplasm*:ti,ab,kw OR tumor*:ti,ab,kw OR tumour*:ti,ab,kw))) AND [embase]/lim | 163,321 |
| #4 | 'patient*':ti,ab,kw AND ('treatment':ti,ab,kw OR 'therapy':ti,ab,kw OR 'monotherapy':ti,ab,kw OR 'adjuvant':ti,ab,kw OR 'neoadjuvant':ti,ab,kw OR 'enrolled':ti,ab,kw OR 'received':ti,ab,kw OR 'treated':ti,ab,kw OR 'assigned':ti,ab,kw OR 'administered':ti,ab,kw) | 7,403,778 |
| #5 | (#1 OR #2) AND #3 AND #4 AND [embase]/lim | 1,855 |

Database(s): Web of Science Core Collection

Executed on 16/09/2025 No date or language filters were used.

| # | Web of Science Core Collection | # hits |
| --- | --- | --- |
| 1 | TS=(("HERE2*" OR "HER-2*" OR "HER/NEU" OR "epidermal growth factor receptor*" OR "EGFR2*" OR "ERBB2*" OR "ERBB-2*" OR "C-ERBB2*" OR "C-ERBB-2*" OR "ERB-B2*" OR "ERB-B-2*" OR "C-ERB-B2*" OR "C-ERB-B-2*" OR "MLN19" OR "MLN-19" OR "p185erbB2" OR "CD340*" OR "CD-340*" OR "NEU" OR "NGL")) | 101,267 |
| 2 | TS=("anti-HER*" OR "antiHER*" OR "Trastuzumab*" OR "Herceptin" OR "R-597" OR "MYL-1401O" OR "CT-P6" OR "SB3" OR "ABP-980" OR "PF-05280014" OR "HLX02" OR "EG12014" OR "Herwenda" OR "Hercessi" OR "Zercepac" OR "Trazimera" OR "Kanjinti" OR "Ontruzant" OR "Credima" OR "Herzuma" OR "Ogivri" OR "Canmab" OR "Hertraz" OR "Pertuzumab" OR "Perjeta" OR "Ado-trastuzumab" OR "Lapatinib" OR "Kadcyla" OR "Tykerb" OR "Neratinib" OR "Nerlynx" OR "Tucatinib" OR "Tukysa" OR "Fam-trastuzumab deruxtecan" OR "Enhertu" OR "Margetuximab" OR "Margenza" OR "Zanidatamab" OR "Ziihera" OR "2C4" OR "Herclon" OR "MM-111" OR "Tyverb" OR "T-DM1" OR "HKI-272" OR "neratinib" OR "Disitamab vedotin") | 44,087 |
| 3 | TS=((bladder OR urothelial OR "upper tract") AND (cancer* OR carcinoma* OR neoplasm* OR tumor* OR tumour*)) | 130,070 |
| 4 | (TI=patient* OR AB=patient*) AND (TI=(treatment OR therapy OR monotherapy OR adjuvant OR neoadjuvant OR enrolled OR received OR treated OR assigned OR administered) OR AB=(treatment OR therapy OR monotherapy OR adjuvant OR neoadjuvant OR enrolled OR received OR treated OR assigned OR administered)) | 4,443,810 |
| 5 | (#1 OR #2) AND #3 AND #4 | 632 |

Registry: Cochrane Central Register of Controlled Trials (CENTRAL; via Ovid)

Executed on 16/09/2025 No date or language filters were used.

| # | CENTRAL | # hits |
| --- | --- | --- |
| 1 | ("HERE2*" or "HER-2*" or "HER/NEU" or "epidermal growth factor receptor*" or "EGFR2*" or "ERBB2*" or "ERBB-2*" or "C-ERBB2*" or "C-ERBB-2*" or "ERB-B2*" or "ERB-B-2*" or "C-ERB-B2*" or "C-ERB-B-2*" or "MLN19" or "MLN-19" or "p185erbB2" or "CD340*" or "CD-340*" or "NEU" or "NGL").mp. [mp=title, original title, abstract, floating sub-heading word, mesh headings, heading words, keyword] | 7931 |
| 2 | ("anti-HER*" or "antiHER*" or "Trastuzumab*" or "Herceptin" or "R-597" or "MYL-1401O" or "CT-P6" or "SB3" or "ABP-980" or "PF-05280014" or "HLX02" or "EG12014" or "Herwenda" or "Hercessi" or "Zercepac" or "Trazimera" or "Kanjinti" or "Ontruzant" or "Credima" or "Herzuma" or "Ogivri" or "Canmab" or "Hertraz" or "Pertuzumab" or "Perjeta" or "Ado-trastuzumab" or "Lapatinib" or "Kadcyla" or "Tykerb" or "Neratinib" or "Nerlynx" or "Tucatinib" or "Tukysa" or "Fam-trastuzumab deruxtecan" or "Enhertu" or "Margetuximab" or "Margenza" or "Zanidatamab" or "Ziihera" or "2C4" or "Herclon" or "MM-111" or "Tyverb" or "T-DM1" or "HKI-272" or "neratinib" or "Disitamab vedotin").mp. [mp=title, original title, abstract, floating sub-heading word, mesh headings, heading words, keyword] | 4782 |
| 3 | 1 or 2 | 10445 |
| 4 | ((bladder or urothelial or "upper tract") and (cancer* or carcinoma* or neoplasm* or tumor* or tumour*)).mp. [mp=title, original title, abstract, floating sub-heading word, mesh headings, heading words, keyword] | 7920 |
| 5 | (patient*).ti,ab. and (treatment or therapy or monotherapy or adjuvant or neoadjuvant or enrolled or received or treated or assigned or administered).ti,ab. | 946841 |
| 6 | 3 and 4 and 5 | 62 |

Search engine: Google Scholar

Executed on 16/09/2025. No date or language filters were used. Top 200 hits were retrieved.

HER2* AND (urothelial OR bladder) AND (cancer OR carcinoma) AND (treatment OR therapy)

Databases(s): SinoMed:

Filters: no language restrictions. The number of results reported in the table includes the use of filters.

Executed on 14/10/2025

| # | Search | # results |
| --- | --- | --- |
| #1 | ( "ErbB受体"[常用字段:智能] OR "HER2"[常用字段:智能] OR "HER-2"[常用字段:智能] OR "HER/NEU"[常用字段:智能] OR "表皮生长因子受体2"[常用字段:智能] OR "EGFR2"[常用字段:智能] OR "酪氨酸激酶受体2"[常用字段:智能] OR "ERBB2"[常用字段:智能] OR "ERBB-2"[常用字段:智能] OR "C-ERBB2"[常用字段:智能] OR "C-ERBB-2"[常用字段:智能] OR "ERB-B2"[常用字段:智能] OR "ERB-B-2"[常用字段:智能] OR "C-ERB-B2"[常用字段:智能] OR "C-ERB-B-2"[常用字段:智能] OR "MLN19"[常用字段:智能] OR "MLN-19"[常用字段:智能] OR "p185erbB2"[常用字段:智能] OR "CD340"[常用字段:智能] OR "CD-340"[常用字段:智能] OR "NEU"[常用字段:智能] OR "NGL"[常用字段:智能]) | 34000 |
| #2 | ( "抗HER2"[常用字段:智能] OR "抗HER-2"[常用字段:智能] OR "HER2靶向"[常用字段:智能] OR "HER-2靶向"[常用字段:智能] OR "靶向HER2"[常用字段:智能] OR "靶向HER-2"[常用字段:智能] OR "曲妥珠单抗"[常用字段:智能] OR "赫赛汀"[常用字段:智能] OR "Trastuzumab"[常用字段:智能] OR "Herceptin"[常用字段:智能] OR "Herclon"[常用字段:智能] OR "R-597"[常用字段:智能] OR "ABP-980"[常用字段:智能] OR "PF-05280014"[常用字段:智能] OR "HLX02"[常用字段:智能] OR "EG12014"[常用字段:智能] OR "Herwenda"[常用字段:智能] OR "Hercessi"[常用字段:智能] OR "Zercepac"[常用字段:智能] OR "汉曲优"[常用字段:智能] OR "Trazimera"[常用字段:智能] OR "Kanjinti"[常用字段:智能] OR "康济尼"[常用字段:智能] OR "Ontruzant"[常用字段:智能] OR "Credima"[常用字段:智能] OR "Herzuma"[常用字段:智能] OR "Ogivri"[常用字段:智能] OR "Canmab"[常用字段:智能] OR "Hertraz"[常用字段:智能] OR "Pertuzumab"[常用字段:智能] OR "Perjeta"[常用字段:智能] OR "2C4"[常用字段:智能] OR "帕妥珠单抗"[常用字段:智能] OR "Ado-trastuzumab emtansine"[常用字段:智能] OR "恩美曲妥珠单抗"[常用字段:智能] OR "Kadcyla"[常用字段:智能] OR "T-DM1"[常用字段:智能] OR "赫赛莱OR Lapatinib"[常用字段:智能] OR "Tyverb"[常用字段:智能] OR "Tykerb OR拉帕替尼"[常用字段:智能] OR "Neratinib"[常用字段:智能] OR "来那替尼"[常用字段:智能] OR "奈拉替尼"[常用字段:智能] OR "Nerlynx"[常用字段:智能] OR "HKI-272"[常用字段:智能] OR "Tucatinib"[常用字段:智能] OR "图卡替尼"[常用字段:智能] OR "妥卡替尼"[常用字段:智能] OR "Tukysa"[常用字段:智能] OR "Fam-trastuzumab deruxtecan"[常用字段:智能] OR "Enhertu"[常用字段:智能] OR "德曲妥珠单抗"[常用字段:智能] OR "优赫得"[常用字段:智能] OR "DS8201"[常用字段:智能] OR "Margetuximab"[常用字段:智能] OR "Margenza "[常用字段:智能] OR "MGAH22"[常用字段:智能] OR "玛格妥昔单抗"[常用字段:智能] OR "Zanidatamab"[常用字段:智能] OR "Ziihera"[常用字段:智能] OR "ZW25"[常用字段:智能] OR "泽尼达妥单抗"[常用字段:智能] OR "MM-111"[常用字段:智能] OR "Disitamab vedotin"[常用字段:智能] OR "RC48"[常用字段:智能] OR "维迪西妥单抗"[常用字段:智能] OR "爱地希"[常用字段:智能]) | 3127 |
| #3 | ( "尿路上皮"[常用字段:智能] OR "上尿路"[常用字段:智能] OR "肾盂"[常用字段:智能] OR "输尿管"[常用字段:智能] OR "下尿路"[常用字段:智能] OR "膀胱"[常用字段:智能] OR "尿道"[常用字段:智能]) AND( "癌"[常用字段:智能] OR "恶性肿瘤"[常用字段:智能]) | 54002 |
| #4 | (#1 OR #2) AND #3 | 336 |

常用字段：由中文标题、摘要、关键词、主题词四个检索项组成。

智能检索：实现检索词及其同义词(含主题词)的扩展检索。

Databases(s): Wanfang:

Filters: no language restrictions. The number of results reported in the table includes the use of filters.

Executed on 14/10/2025

| # | Search | # results |
| --- | --- | --- |
| #1 | "ErbB受体" OR "HER2" OR "HER-2" OR "HER/NEU" OR "表皮生长因子受体2" OR "EGFR2" OR "酪氨酸激酶受体2" OR "ERBB2" OR "ERBB-2" OR "C-ERBB2" OR "C-ERBB-2" OR "MLN19" OR "p185erbB2" OR "CD340" | 74662 |
| #2 | "抗HER2" OR "抗HER-2" OR "HER2靶向" OR "HER-2靶向" OR "靶向HER2" OR "靶向HER-2" OR "曲妥珠单抗" OR "赫赛汀" OR "Trastuzumab" OR "Herceptin" OR "汉曲优" OR "Pertuzumab" OR "Perjeta" OR "2C4" OR "帕妥珠单抗" OR "Ado-trastuzumab emtansine" OR "恩美曲妥珠单抗" OR "T-DM1" OR "赫赛莱" OR "Lapatinib" OR "拉帕替尼" OR "Neratinib" OR "来那替尼" OR "奈拉替尼" OR "Tucatinib" OR "图卡替尼" OR "Fam-trastuzumab deruxtecan" OR "Enhertu" OR "德曲妥珠单抗" OR "DS8201" OR "Margetuximab" OR "玛格妥昔单抗" OR "Zanidatamab" OR "泽尼达妥单抗" OR "Disitamab vedotin" OR "RC48" OR "维迪西妥单抗" OR "爱地希" | 27282 |
| #3 | (("尿路上皮" OR "上尿路" OR "肾盂" OR "输尿管" OR "下尿路" OR "膀胱" OR "尿道") AND ("癌" OR "恶性肿瘤")) | 267317 |
| #4 | (#1 OR #2) AND #3 | 369 |

检索表达式（中英文扩展&主题词扩展）：(("ErbB受体" OR "HER2" OR "HER-2" OR "HER/NEU" OR "表皮生长因子受体2" OR "EGFR2" OR "酪氨酸激酶受体2" OR "ERBB2" OR "ERBB-2" OR "C-ERBB2" OR "C-ERBB-2" OR "MLN19" OR "p185erbB2" OR "CD340") OR ("抗HER2" OR "抗HER-2" OR "HER2靶向" OR "HER-2靶向" OR "靶向HER2" OR "靶向HER-2" OR "曲妥珠单抗" OR "赫赛汀" OR "Trastuzumab" OR "Herceptin" OR "汉曲优" OR "Pertuzumab" OR "Perjeta" OR "2C4" OR "帕妥珠单抗" OR "Ado-trastuzumab emtansine" OR "恩美曲妥珠单抗" OR "T-DM1" OR "赫赛莱" OR "Lapatinib" OR "拉帕替尼" OR "Neratinib" OR "来那替尼" OR "奈拉替尼" OR "Tucatinib" OR "图卡替尼" OR "Fam-trastuzumab deruxtecan" OR "Enhertu" OR "德曲妥珠单抗" OR "DS8201" OR "Margetuximab" OR "玛格妥昔单抗" OR "Zanidatamab" OR "泽尼达妥单抗" OR "Disitamab vedotin" OR "RC48" OR "维迪西妥单抗" OR "爱地希")) AND (("尿路上皮" OR "上尿路" OR "肾盂" OR "输尿管" OR "下尿路" OR "膀胱" OR "尿道") AND ("癌" OR "恶性肿瘤"))

Databases(s): China National Knowledge Infrastructure (CNKI):

Filters: no language restrictions. The number of results reported in the table includes the use of filters.

Executed on 14/10/2025

| # | Search | # results |
| --- | --- | --- |
| #1 | TKA=('ErbB受体' + 'HER2' + 'HER-2' + 'HER/NEU' + '表皮生长因子受体2' + 'EGFR2' + '酪氨酸激酶受体2' + 'ERBB2' + 'ERBB-2' + 'C-ERBB2' + 'C-ERBB-2' + 'ERB-B2' + 'ERB-B-2' + 'C-ERB-B2' + 'C-ERB-B-2' + 'MLN19' + 'MLN-19' + 'p185erbB2' + 'CD340' + 'CD-340' + 'NEU' + 'NGL') | 35794 |
| #2 | TKA=('抗HER2' + '抗HER-2' + 'HER2靶向' + 'HER-2靶向' + '靶向HER2' + '靶向HER-2' + '曲妥珠单抗' + '赫赛汀' + 'Trastuzumab' + 'Herceptin' + 'Herclon' + 'R-597' + 'ABP-980' + 'PF-05280014' + 'HLX02' + 'EG12014' + 'Herwenda' + 'Hercessi' + 'Zercepac' + '汉曲优' + 'Trazimera' + 'Kanjinti' + '康济尼' + 'Ontruzant' + 'Credima' + 'Herzuma' + 'Ogivri' + 'Canmab' + 'Hertraz' + 'Pertuzumab' + 'Perjeta' + '2C4' + '帕妥珠单抗' + 'Ado-trastuzumab emtansine' + '恩美曲妥珠单抗' + 'Kadcyla' + 'T-DM1' + '赫赛莱' + 'Lapatinib' + 'Tyverb' + 'Tykerb' + '拉帕替尼' + 'Neratinib' + '来那替尼' + '奈拉替尼' + 'Nerlynx' + 'HKI-272' + 'Tucatinib' + '图卡替尼' + '妥卡替尼' + 'Tukysa' + 'Fam-trastuzumab deruxtecan' + 'Enhertu' + '德曲妥珠单抗' + '优赫得' + 'DS8201' + 'Margetuximab' + 'Margenza' + 'MGAH22' + '玛格妥昔单抗' + 'Zanidatamab' + 'Ziihera' + 'ZW25' + '泽尼达妥单抗' + 'MM-111' + 'Disitamab vedotin' + 'RC48' + '维迪西妥单抗' + '爱地希') | 9157 |
| #3 | TKA=('尿路上皮' + '上尿路' + '肾盂' + '输尿管' + '下尿路' + '膀胱' + '尿道')*('癌' + '恶性肿瘤') | 24154 |
| #5 | (#1 OR #2) AND #3 | 238 |

资源范围：总库; 同义词扩展; 时间范围：更新时间：不限；

详细检索式：TKA=((('ErbB受体' + 'HER2' + 'HER-2' + 'HER/NEU' + '表皮生长因子受体2' + 'EGFR2' + '酪氨酸激酶受体2' + 'ERBB2' + 'ERBB-2' + 'C-ERBB2' + 'C-ERBB-2' + 'ERB-B2' + 'ERB-B-2' + 'C-ERB-B2' + 'C-ERB-B-2' + 'MLN19' + 'MLN-19' + 'p185erbB2' + 'CD340' + 'CD-340' + 'NEU' + 'NGL')+('抗HER2' + '抗HER-2' + 'HER2靶向' + 'HER-2靶向' + '靶向HER2' + '靶向HER-2' + '曲妥珠单抗' + '赫赛汀' + 'Trastuzumab' + 'Herceptin' + 'Herclon' + 'R-597' + 'ABP-980' + 'PF-05280014' + 'HLX02' + 'EG12014' + 'Herwenda' + 'Hercessi' + 'Zercepac' + '汉曲优' + 'Trazimera' + 'Kanjinti' + '康济尼' + 'Ontruzant' + 'Credima' + 'Herzuma' + 'Ogivri' + 'Canmab' + 'Hertraz' + 'Pertuzumab' + 'Perjeta' + '2C4' + '帕妥珠单抗' + 'Ado-trastuzumab emtansine' + '恩美曲妥珠单抗' + 'Kadcyla' + 'T-DM1' + '赫赛莱' + 'Lapatinib' + 'Tyverb' + 'Tykerb' + '拉帕替尼' + 'Neratinib' + '来那替尼' + '奈拉替尼' + 'Nerlynx' + 'HKI-272' + 'Tucatinib' + '图卡替尼' + '妥卡替尼' + 'Tukysa' + 'Fam-trastuzumab deruxtecan' + 'Enhertu' + '德曲妥珠单抗' + '优赫得' + 'DS8201' + 'Margetuximab' + 'Margenza' + 'MGAH22' + '玛格妥昔单抗' + 'Zanidatamab' + 'Ziihera' + 'ZW25' + '泽尼达妥单抗' + 'MM-111' + 'Disitamab vedotin' + 'RC48' + '维迪西妥单抗' + '爱地希'))*(('尿路上皮' + '上尿路' + '肾盂' + '输尿管' + '下尿路' + '膀胱' + '尿道')*('癌' + '恶性肿瘤')))
