# Supplementary material for: Efficacy of HER2-targeted therapies in localized urothelial carcinoma: a systematic review and meta-analysis
Source: World J Urol. 2026 Nov 14;44(1):810. doi: 10.1007/s00345-026-06868-2 (PMC13769737; doi:10.1007/s00345-026-06868-2)
Supplement: Supplementary file 6 [file 345_2026_6868_MOESM5_ESM.docx]

**Identification of studies via databases and registers**

**Identification of studies via databases - update**

**Identification of studies via databases and registers**

Records identified from:

Databases (n = 943)

- Sinomed (n = 336)

- CNKI (n = 238)

- Wanfang (n = 369)

Records removed before screening:

Duplicate records removed

(n = 323)

Records screened

(n = 620)

Reports sought for retrieval

(n = 18)

Reports not retrieved

(n = 0)

Reports assessed for eligibility (n = 18)

Reports excluded:

- Wrong population (n = 6)

- Wrong study design (n = 4)

- Cohort duplicate (n = 2)

- Wrong outcome (n = 2)

Records excluded**

(n = 602)

Records identified from*:

Databases (n = 384)

- Pubmed (n = 65)

- Embase (n = 147)

- Web of Science (n = 167)

- CENTRAL (n = 5)

Records removed *before screening*:

Duplicate records removed (n = 126)

Records screened

(n = 258)

Reports sought for retrieval

(n = 56)

Reports not retrieved

(n = 0)

Reports assessed for eligibility (n = 56)

Reports excluded:

- Wrong study design (n = 22)

- Cohort duplicate (n = 2)

- Wrong population (n = 15)

- Wrong outcome (n = 12)

Records removed *before screening*:

Duplicate records removed (n = 1296)

Records identified from*:

Databases (n = 3374)

- Pubmed (n = 625)

- Embase (n = 1855)

- Web of Science (n = 632)

- CENTRAL (n = 62)

- Google Scholar (n = 200)

**Identification**

Records excluded**

(n = 202)

Records excluded**

(n = 1903)

Records screened

(n = 2078)

Reports sought for retrieval

(n = 175)

Reports not retrieved

(n = 0)

**Screening**

Reports excluded:

- Wrong study design (n = 46)

- Cohort duplicate (n = 40)

- Wrong population (n = 42)

- Wrong outcome (n = 23)

Reports assessed for eligibility (n = 175)

Studies included in review

(n = 33)

Reports of included studies

(n = 33)

**Included**

Source: Page MJ, et al. BMJ 2021;372:n71. doi: 10.1136/bmj.n71.

This work is licensed under CC BY 4.0. To view a copy of this license, visit <https://creativecommons.org/licenses/by/4.0/>
